# Additive-Free Reductive Cleavage of Lignin Model Compounds by Ethanol-Sensitized Titania under Visible Light: A Sustainable Approach to β‑O‑4 Bond Cleavage

**DOI:** 10.1021/acssuschemeng.5c03068

**Published:** 2025-11-06

**Authors:** Ayesha Khan, Logan W. Evans, David B. C. Martin

**Affiliations:** Department of Chemistry, 4083 University of Iowa, Iowa City, Iowa 52242, United States

**Keywords:** ligand-to-metal charge transfer complex, β-O-4 ketone, lignin, nitrogen doping, hydroxyl groups

## Abstract

The selective cleavage of the β-O-4 bond in lignin model compounds is a crucial step in realizing the full potential of transforming lignin to value-added aromatic compounds. Herein, we report an additive-free, visible-light-driven ligand-to-metal charge transfer (LMCT)-mediated strategy for the reductive cleavage of β-O-4 lignin model ketones in ethanol, utilizing TiO_2_ as the photocatalyst. We found that ethanol can act as a sensitizer and forms an LMCT complex on the surface of titania, which enables the reductive cleavage of the β-O-4 ketone under blue light (440 nm). The LMCT-sensitized titania showed moderate conversion (58%) for the reductive cleavage of β-O-4 ketone. Additionally, we observed that nitrogen doping of titania further enhances the visible light absorption and improves the conversion of the β-O-4 ketone up to 91%. EPR studies indicate the in situ generation of Ti^3+^ under blue light irradiation, which likely enables the reductive cleavage of β-O-4 ketone. Our work demonstrates a promising potential for the reductive cleavage of β-O-4 lignin model ketone to form value-added aromatic compounds under mild conditions without the use of an additional reductant by using ethanol (reaction solvent).

## Graphical abstract

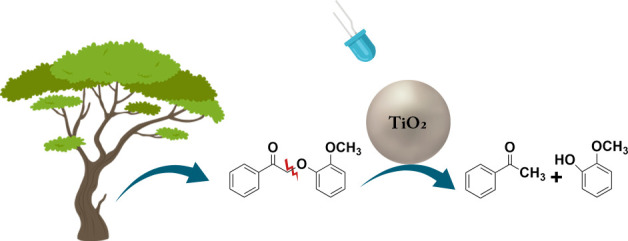

## Introduction

Lignin valorization offers a sustainable way to produce industrially relevant aromatic compounds. The key challenge in valorization of lignin is the selective scission of the β-O-4 bond, which accounts for 43–65% of all linkages present in lignin.^1–3^ Owing to the complex structure, diverse functional groups, and limited solubility in common solvents, the direct transformation of naturally sourced lignin molecules via the selective cleavage of the β-O-4 bond is quite challenging.^4–7^ Lignin model compounds containing β-O-4 bonds have been widely investigated to study the selective cleavage of aryl ether linkages to produce valuable aromatic compounds.^8–12^ This provides insights into the reactivity of specific functional groups and mechanisms of β-O-4 bond cleavage in lignin.

Conventional thermocatalytic routes have been extensively explored for the selective scission of the β-O-4 bond in lignin model compounds. However, this approach requires precious metal catalysts,^13,14^ high temperature (65–260 °C),^13,15–17^ and high H_2_ pressure (≥10 bar).^13,18^ Elevated pressure can result in the hydrogenation of aromatic rings and undesired side products.^13^ Moreover, the need for expensive catalysts and energy-intensive conditions makes this approach economically less viable on a large scale and raises concerns about the sustainability of the overall process. The development of mild, low-temperature, and highly selective catalytic methods for the selective scission of β-O-4 bonds in lignin model compounds is of great significance from a sustainability viewpoint.

Compared to thermocatalytic approaches, photocatalytic methods are advantageous due to the mild conditions, low temperature, and high efficiency. Stephenson and co-workers developed a photocatalytic method for the reductive cleavage of β-O-4 model ketone compounds using [Ir­(ppy)_2_(dtbbpy)]­PF_6_ under blue light_._ The iridium-based photocatalyst effectively promotes the C–O bond cleavage and forms the desired fragmentation products in high yields (up to 95%).^19^ However, the high cost of the iridium-based catalyst makes this approach less economical on a large scale. A heterogeneous photocatalytic approach with mild conditions and a low-cost photocatalyst is a promising strategy to drive the conversion of lignin model compounds.^8,20–23^ Wang and co-workers employed low-cost TiO_2_ as a photocatalyst to accomplish the selective scission of β-O-4 model ketones (Scheme 1A) in the presence of sodium acetate in ethanol under UV light (365 nm).^24^ This strategy gives good to high yields (up to 93%) of fragmentation products. However, high-energy UV light poses safety issues and raises concerns about the overall desirability of the reaction.

**Scheme 1:**
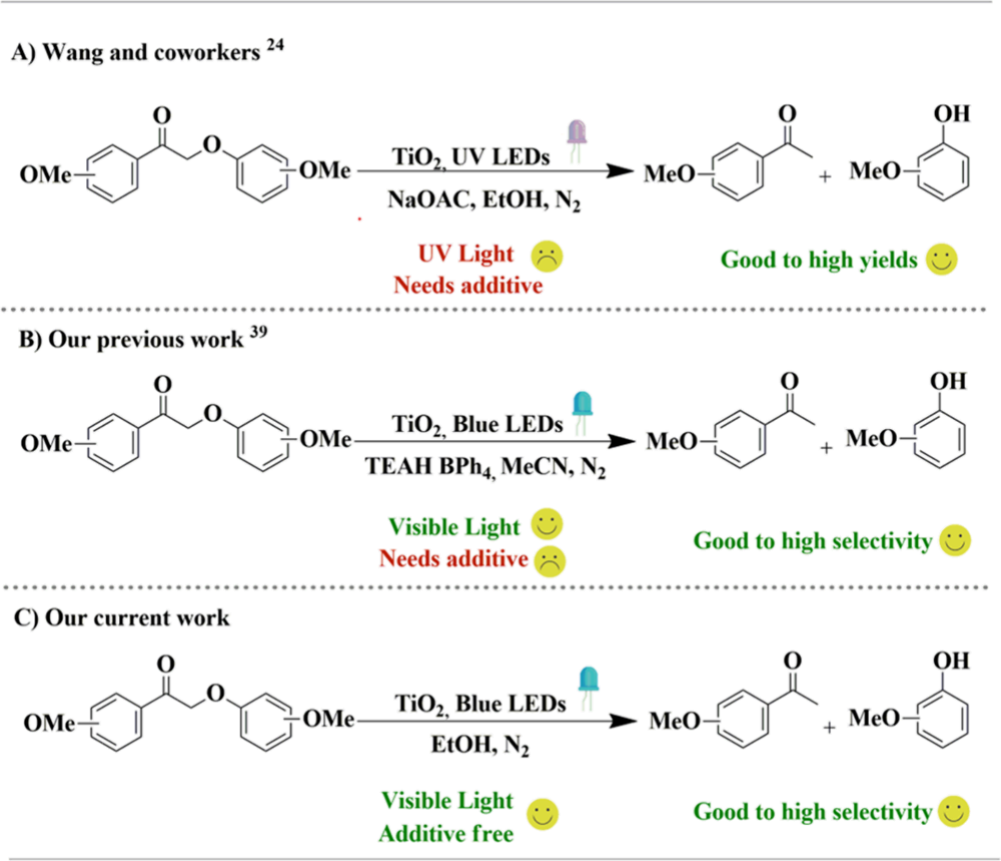
(A) TiO_2_-Catalyzed Reductive Cleavage of β-O-4 Lignin Model Ketones under UV Light; (B) Reductive Cleavage of β-O-4 Lignin Model Ketones by Triethylamine-Sensitized TiO_2_ under Blue Light; (C) Reductive Cleavage of β-O-4 Lignin Model Ketones by Ethanol-Sensitized TiO_2_ under Blue Light

Titania typically absorbs UV light due to its wide band gap (∼3.2 eV). To make the TiO_2_-catalyzed reactions more attractive, the optical response of titania can be modified to absorb visible light. Strategies to improve the visible light response of titania include metal or nonmetal doping of titania,^25–27^ coupling titania with other narrow-bandgap semiconductors,^28^ dye-sensitization,^29^ and ligand-to-metal charge transfer (LMCT) sensitization.^30^ Nitrogen doping has been widely investigated to improve the visible light absorption of titania by narrowing the band gap and/or the creation of oxygen vacancies,^31^ which is cost-effective due to readily available and low-cost nitrogen sources (urea, alkyl amine, etc.), which can be used as a nitrogen dopant. LMCT sensitization involves the sensitization of titania by adsorbates that form a complex on the surface of titania and induce visible light absorption. Upon visible light irradiation, electrons are transferred from the surface complex to the conduction band of titania. It was found that organic compounds with hydroxyl groups (benzyl alcohol,^32^ catechol,^33^ phenol,^34^ glucose,^35^ hydroxymethylfurfural,^36^ etc.), amine group (indole),^37^ and carboxylic acid groups (formic acid)^38^ can form an LMCT complex on the surface of titania and introduce a new absorption band in the visible light region.

Our previous work showed that β-O-4 lignin model alcohols can act as LMCT sensitizers for titania and enable the oxidation of the alcohols to β-O-4 ketones under green light (525 nm).^39^ Additionally, we found that triethylammonium tetraphenylborate and hexafluorophosphate salts that are used as proton donors can form LMCT complexes on the surface of titania via the adsorption of triethylamine and induce visible light absorption. This provides a sustainable pathway for the reductive cleavage of β-O-4 ketone under blue light in acetonitrile (Scheme 1B).^39^ To make the TiO_2_-catalyzed reductive cleavage of the β-O-4 ketone more economical and greener, we aimed to develop a method that does not require an additional reductant or sensitizer to accomplish the reductive cleavage of the β-O-4 ketone under visible light. Based on examples from the literature^30^ and our previous work,^39^ we hypothesized that an alcohol such as ethanol could form an LMCT complex with titania and improve its visible light absorption, serving as both the sensitizer and the reductant to achieve the reductive cleavage of β-O-4 ketone under visible light. Additionally, we proposed that nitrogen doping could enable reductive cleavage under visible light. Herein, we show that nitrogen doping and LMCT sensitization of titania by ethanol enable the efficient and selective cleavage of β-O-4 lignin model ketones under mild conditions without the need of an additional reductant, which is beneficial for the advancement of sustainable catalysis.

## Experimental Section

### Synthesis of Undoped Titania Nanoparticles

Titania nanoparticles were synthesized via sol–gel method followed by hydrothermal treatment.^39^ The detailed synthesis of undoped titania is mentioned in the Supporting Information.

### Synthesis of Nitrogen-Doped Titania Nanoparticles

Nitrogen-doped titania nanoparticles were synthesized using different nitrogen dopant sources (urea, iso-propylamine, and *n*-butylamine) by the hydrothermal method adapted from the literature.^40–42^ To synthesize nitrogen-doped titania using urea as a nitrogen dopant, 0.01252 mol of Ti­(Oi-Pr)_4_ was dissolved in 25 mL of 2-propanol under continuous stirring. Then, 0.0375 mol of urea was dissolved in 40 mL of water (acidified with 1 mL of 1 M nitric acid). Subsequently, the acidified urea solution was added slowly to the Ti­(Oi-Pr)_4_ and propanol mixture under vigorous stirring (400 rpm), until a gel was formed. The gel was stirred for an additional 3 h and then transferred to a Teflon-lined autoclave for hydrothermal treatment at 200 °C for 24 h in an oven. The obtained nitrogen-doped titania nanoparticles were filtered, washed several times with deionized water, and dried at 110 °C for 24 h. The prepared nitrogen-doped sample was named N-SGHT-U(1:3). The nitrogen-doped titania nanoparticles were also prepared with 1:1 and 1:5 molar ratios of Ti­(Oi-Pr)_4_ to urea (U). The as-synthesized samples were denoted as N-SGHT:U(1:1) and N-SGHT:U(1:5), respectively. The name of the samples was assigned according to the molar ratio of Ti­(Oi-Pr)_4_ to urea. Next, nitrogen-doped titania nanoparticles were synthesized using iso-propylamine (IPA) and *n*-butylamine (NBA) as the nitrogen dopant source, following the same procedure. The as-prepared samples were named N-SGHT:IPA(1:3) and N-SGHT:NBA(1:3), respectively. The nitrogen doping of commercial titania was carried out by adding 1g of commercial titania P25 to 70 mL of acidified urea (0.0375 mol) solution and stirring for 3 h; the suspension was then transferred to a Teflon-lined autoclave for hydrothermal treatment at 200 °C for 24 h in an oven. The obtained nitrogen-doped commercial titania was filtered and dried at 110 °C for 24 h. The as-prepared sample was denoted as N-P25-U(1:3).

### Characterization

The details regarding the methods adopted for the catalyst characterization and synthesis of β-O-4 lignin model ketone substrates are provided in the Supporting Information.

### Photocatalytic Activity Test

#### Photocatalytic Reductive Cleavage of β-O-4 Lignin Model Ketone

Photocatalytic reductive cleavage of the β-O-4 lignin model ketone was performed in a glass vial. Typically, 15 mL of 0.5 mM β-O-4 ketone (0.0075 mmol) solution in ethanol and 45 mg of SGHT-200 were loaded into the glass vial. The vial was covered with a septum (14 × 20, VWR), sealed with a parafilm, and placed into the Hepato Chem, EvoluChemTM PhotoRedOx Box (Figure S1). The suspension was stirred for 1 h in the dark to establish an equilibrium. The reaction mixture was then continuously bubbled with N_2_ under blue light (Kessil PR-160L 440 nm) for 6 h. A built-in fan kept the reaction conditions at room temperature. The average intensity of the Kessil PR-160L 440 nm lamp measured from 1 cm distance was 399 mW/cm^2^. Moreover, the PhotoRedOx Box was equipped with mirrors to enhance light distribution. The aliquots were collected and filtered by using 0.22 μm nylon syringe filters to remove the catalyst. After the reaction, the used catalyst was collected, washed twice with acetonitrile and water, dried at 110 °C, and then used in the next reaction using fresh substrate.

The reactants and products were analyzed by HPLC using a Shimadzu LC2040C equipped with a Thermo Scientific C18 Column (3 mm diameter, 100 mm length). The flow rate was set at 0.4 mL/min, and the oven temperature was kept at 40 °C. The mobile phase was used for the quantitative analysis of β-O-4 lignin model ketones, and the conversion products were acetonitrile and water with a volume ratio of 55:45. The conversion and selectivity were calculated in the liquid phase according to the following formulas:

$$\mathrm{Conversion}\%=\frac{S_{\mathrm{init}.}-S_{\mathrm{final}}}{S_{\mathrm{init}.}}\times 100$$ 1

$$\mathrm{Selectivity}\%=\frac{P}{S_{\mathrm{init}.}-S_{\mathrm{final}}}\times 100$$ 2

where *S*_init_ refers to the initial amount of substrate (in mmoles), *S*_final_ corresponds to the amount of substrate after the reaction (in mmoles), and *P* is the product formed after the reaction (in mmoles).

The photocatalytic cleavage of ethanosolv lignin was performed in a similar way. Complete experimental details are provided in the Supporting Information.

## Results and Discussion

### Characterization of Undoped and Nitrogen-Doped Titania

XRD analysis reveals that SGHT-200 is composed of anatase and brookite phases (Figure S2) in the ratio of 79:21 (entry 1, Table 1). We found that when urea was used as a nitrogen dopant source, the percentage of the brookite phase was slightly (∼10%) reduced (entries 2–4, Table 1). In contrast, when *n*-butylamine (N-SGHT-NBA(1:3)) and iso-propylamine (N-SGHT-IPA(1:3)) were used as nitrogen dopants, only the anatase phase was found (entries 5 and 6, Table 1). The nitrogen doping may induce strain in the lattice and result in the generation of oxygen vacancies, both of which can cause displacement of the Ti atom. This may result in the transformation of the unstable brookite phase to the more stable anatase phase.^43^ Moreover, N-SGHT-NBA(1:3) and N-SGHT-IPA(1:3) exhibited larger crystal sizes (entries 5 and 6, Table 1) than SGHT-200 and nitrogen-doped titania samples prepared using urea. Tavares and co-workers reported that the unreacted nitrogen dopant source could hinder the formation of smaller crystals.^44^ However, the IR spectra of the nitrogen-doped titania samples lack any strong band associated with nitrogen dopant sources (Figure S3). The N-SGHT-NBA(1:3) showed very weak bands around 1059 and 955 cm^–1^, which could be related to the C–N stretching vibration of *n*-butylamine. P25 and N-P25-U(1:3) were found to be composed of anatase and rutile phases (Figure S4).

**Table 1:** Crystallographic Features and Textural Properties (Brunauer–Emmett–Teller Specific Surface Area [SSA], Barrett–Joyner–Halenda [BJH] Pore Volume, and Pore Radius) of Undoped and Nitrogen-Doped Titania Photocatalysts^a^

		ratio of crystalline phases (%)	crystal size (nm)					
entry	catalyst	anatase:brookite:rutile	anatase	brookite	rutile	SSA (m^2^ g^–1^)	BJH pore radius (Å)	BJH pore volume (cm^3^ g^–1^)
1	SGHT-200	79:21:0	11	8	NA	109	55	0.335
2	N-SGHT-U(1:1)	89:11:0	14	8	NA	96	38	0.214
3	N-SGHT-U(1:3)	91:9:0	13	7	NA	103	50	0.313
4	N-SGHT-U(1:5)	90:10:0	10	6	NA	121	51	0.255
5	N-SGHT-IPA(1:3)	100:0:0	30	NA	NA	37	111	0.229
6	N-SGHT-NBA(1:3)	100:0:0	27	NA	NA	40	93	0.242
7	P25	87:0:13	25	NA	42	46	171	0.465
8	N-P25-U (1:3)	85:0:15	28	NA	37	35	111	0.421

a NA: not applicable.

XPS analysis shows that the Ti 2p core level spectrum of SGHT-200 is deconvoluted into two peaks (Figure 1a) appearing at 458.6 and 464.3 eV, attributed to Ti^4+^ 2p_3/2_ and Ti^4+^ 2p_1/2_, respectively, while the Ti 2p spectrum of N-SGHT-U(1:3) showed the presence of Ti^4+^, Ti^3+^, and Ti^2+^ (Figure 1a), at binding energies of 458.6, 457.2, and 455.4 eV, respectively. N-SGHT-NBA(1:3) also showed the presence of Ti^3+^ and Ti^2+^ in addition to Ti^4+^ (Figure S6). However, no Ti^3+^ and Ti^2+^ was detected (Figure S6) in N-SGHT-IPA(1:3) and N-P25-U(1:3).

The undoped titania (SGHT-200) and nitrogen-doped titania samples prepared using urea as a nitrogen dopant exhibit a type IV isotherm with an H2 hysteresis loop (Figure S5), which is characteristic of mesoporous materials with ink-bottle shaped pores.^45^ However, the nitrogen-doped titania sample prepared using iso-propylamine and *n*-butylamine showed a type II isotherm with an H3 hysteresis loop (Figure S5), which is indicative of mesoporous materials with wedge-shaped pores.^45^ We found that the undoped titania (SGHT-200) exhibited a high specific surface area (109 m^2^ g^–1^) and the nitrogen-doped titania prepared using 1:1 and 1:3 molar ratios of Ti­(Oi-Pr)_4_ to urea exhibited similar specific surface areas (entries 2 and 3, Table 1). However, when the molar ratio of Ti­(Oi-Pr)_4_ to urea was 1:5, a slight increase in the specific surface area (121 m^2^ g^–1^) was observed, possibly due to the thermal decomposition of urea to CO_2_ bubbles.^46,47^ Additionally, Chi et al. observed that urea as a nitrogen dopant leads to higher nucleation sites and slower crystal growth, which results in a smaller crystal size and higher specific surface area of N-doped mesoporous titania spheres.^46^ Similarly, we also observed a relatively smaller crystal size and higher specific surface area of titania at a higher molar ratio of urea (entry 4, Table 1). However, the nitrogen-doped titania samples prepared using *iso*-propylamine and *n*-butylamine showed a much smaller specific surface area (entries 5 and 6, Table 1). This could be related to the adsorption of unreacted alkyl amines on the surface of titania, which may block the pores and decrease the surface area.

XPS analysis shows that the Ti 2p core level spectrum of SGHT-200 is deconvoluted into two peaks (Figure 1a) appearing at 458.6 and 464.3 eV, attributed to Ti^4+^ 2p_3/2_ and Ti^4+^ 2p_1/2_, respectively, while the Ti 2p spectrum of N-SGHT-U(1:3) showed the presence of Ti^4+^, Ti^3+^, and Ti^2+^ (Figure 1a), at binding energies of 458.6, 457.2, and 455.4 eV, respectively. N-SGHT-NBA(1:3) also showed the presence of Ti^3+^ and Ti^2+^ in addition to Ti^4+^ (Figure S6). However, no Ti^3+^ and Ti^2+^ was detected (Figure S6) in N-SGHT-IPA(1:3) and N-P25-U(1:3).

The O 1s spectrum of SGHT-200 showed two main peaks; the peak observed at 530.3 eV (Figure 1b) corresponds to the Ti–O bond in the titania lattice,^48,49^ and the signal appearing at 531.2 eV (Figure 1b) is indexed to Ti–OH bonds in titania.^49^ The nitrogen-doped titania samples also exhibited signals related to Ti–OH bonds in addition to lattice Ti–O bonds (Figures 1b and S7). The N 1s spectrum of N-SGHT-U(1:3) exhibits a peak at a binding energy of 398.3 eV (Figure 1c), which may be attributed to the interstitial nitrogen incorporation in the titania structure.^48^ Similarly, N-SGHT-NBA(1:3) and N-P25-U(1:3) also exhibit signals related to nitrogen doping at 398.9 eV (Figure S8).

**Figure 1:**
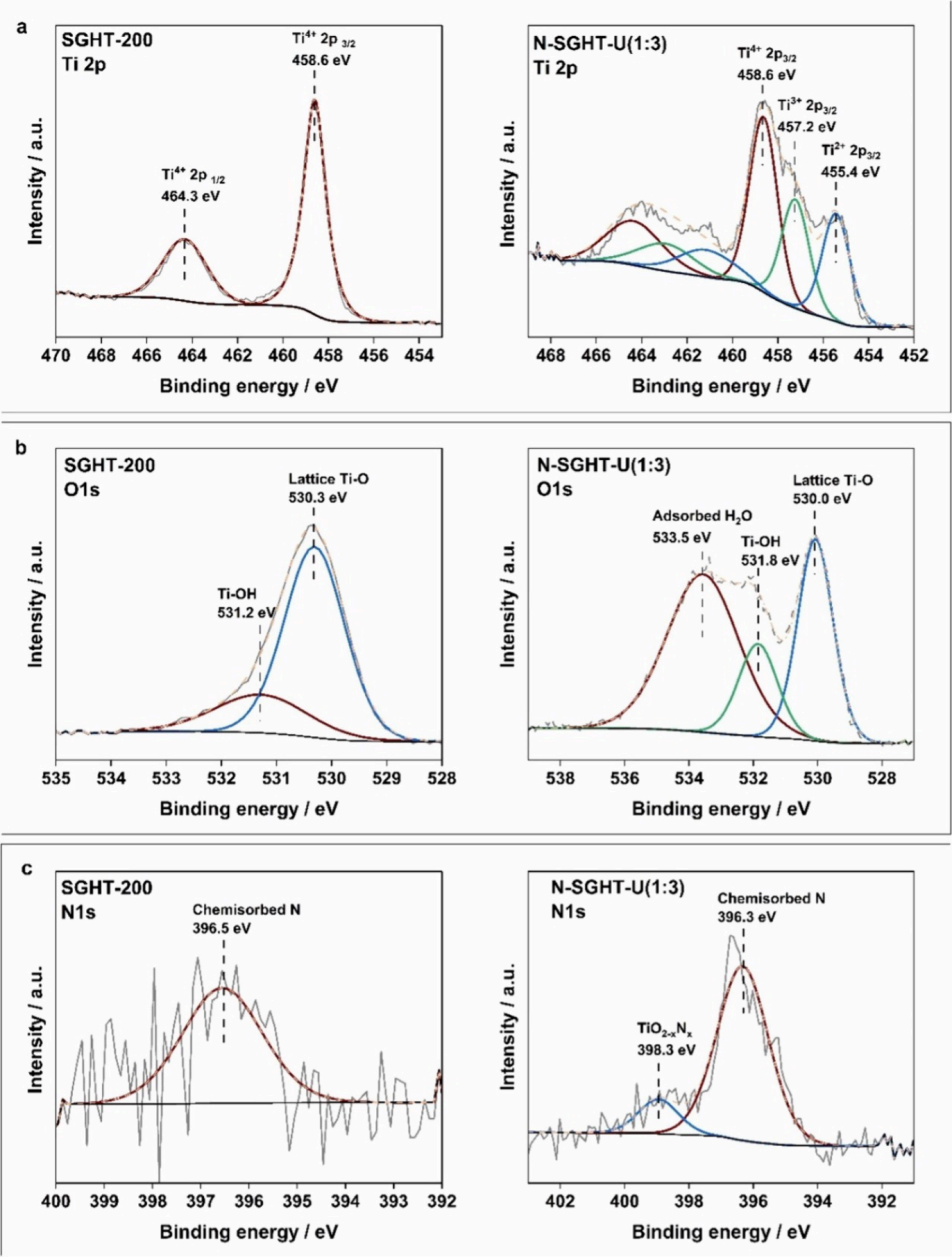
XPS spectra: (a) Ti 2p, (b) O 1s, and (c) N 1s spectra of undoped and nitrogen-doped titania photocatalysts.

The DRS UV–visible absorption spectrum of undoped titania (SGHT-200) showed absorption in the UV region, which is characteristic of pristine titania (Figure 2a). The nitrogen doping of titania using different nitrogen dopant sources slightly enhanced the absorption in the visible range of 420–500 nm (Figure 2a). The band gap of SGHT-200 estimated through the Kubelka–Munk function was ∼3.2 eV (Figure 2b), while the nitrogen-doped titania sample showed a band gap of ∼3.1 eV (Figure 2b). It has been observed that the nitrogen doping of titania did not change the band gap of titania noticeably, which may be ascribed to interstitial nitrogen doping. The nitrogen-doped commercial titania P25 showed a marked increase in the absorption of visible light upon nitrogen doping (Figure 3a). Moreover, the band gap of commercial titania P25 reduced to ∼2.9 eV upon nitrogen doping (Figure 3b).

**Figure 2:**
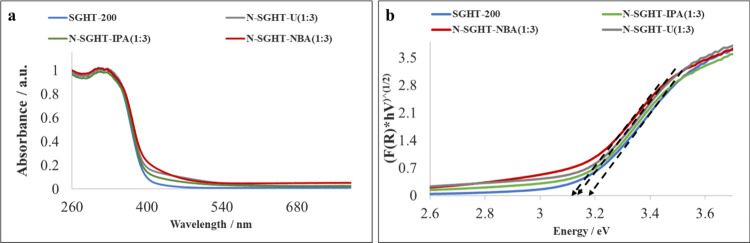
(a) DRS UV–visible absorption spectra of undoped and nitrogen-doped titania photocatalysts. (b) Tauc plots of undoped and nitrogen-doped titania photocatalysts.

**Figure 3:**
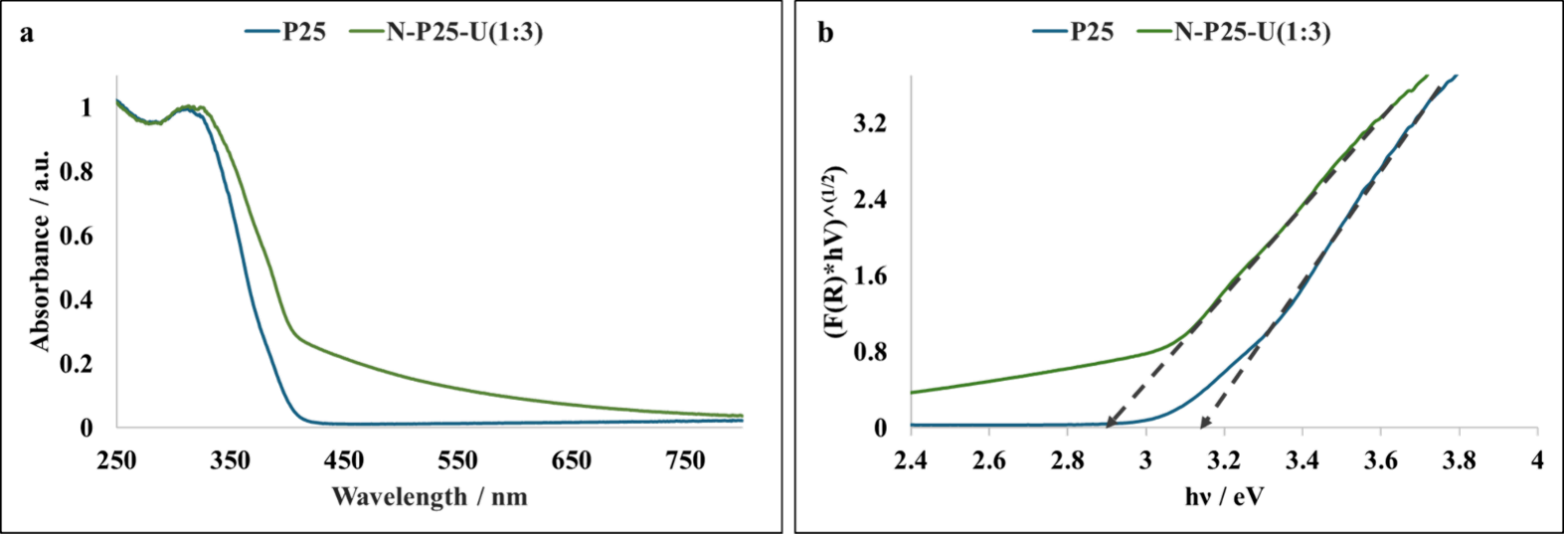
(a) DRS UV–visible absorption spectra of undoped and nitrogen-doped commercial titania P25. (b) Tauc plots of undoped and nitrogen-doped commercial titania.

The transmission electron microscopy (TEM) images showed that the undoped and nitrogen-doped titania nanoparticles were highly aggregated and exhibited variable sizes and shapes (Figure 4a,b). SGHT-200 and N-SGHT-U(1:3) showed similar particle sizes (Figure 4c,d), with average particle sizes of 11 and 12 nm, respectively. However, N-SGHT-IPA(1:3) and N-SGHT-NBA(1:3) exhibit comparatively greater particle sizes, i.e., 34 and 28 nm, respectively (Figures S9 and S10).

**Figure 4:**
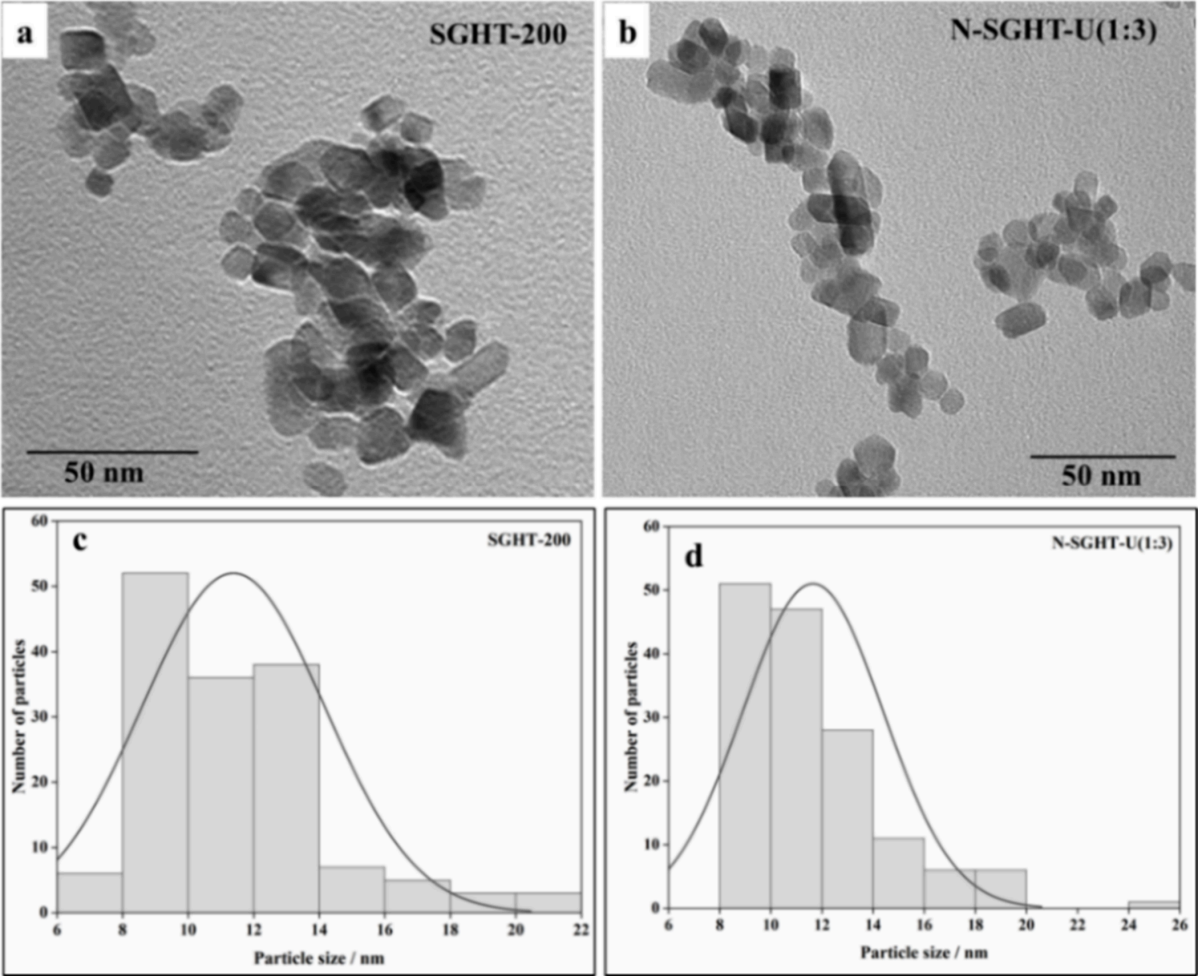
High-resolution TEM images of (a) SGHT-200 and (b) N-SGHT-U(1:3). Particle size distribution in (c) SGHT-200 and (d) N-SGHT-U(1:3).

### Reductive Cleavage of β-O-4 Ketone

We investigated the activity of LMCT-sensitized SGHT-200 for the reductive cleavage of β-O-4 ketone, and a photocatalytic experiment was carried out for the reductive cleavage of model β-O-4 ketone, 2-(2-methoxyphenoxy)-1-phenylethanone (PPEn), to guaiacol and acetophenone under blue light (440 nm). We observed that LMCT sensitization of SGHT-200 by ethanol showed a good conversion for the reductive cleavage of PPEn (entry 2, Table 2). After 6 h of irradiation under blue light, 58% PPEn conversion was noted with high selectivity for acetophenone (84%) and guaiacol (74%) with an optimized 3 g/L catalyst loading (Figure S11). Commercial titania (P25), which is often considered as a reference catalyst in heterogeneous photocatalysis, was also tested for the reductive cleavage of PPEn under blue light. Commercial titania (P25) also showed high selectivity for acetophenone and guaiacol (entry 3, Table 2); however, the activity of P25 was substantially low (20% PPEn conversion) for this reductive cleavage reaction. The reduced activity of P25 may be related to the lower specific surface area of P25 (46 m^2^ g^–1^) than that of SGHT-200 (109 m^2^ g^–1^). Prior studies carried out on the sensitization of titania via an LMCT complex formation demonstrated that the specific surface area of titania significantly influences the activity of LMCT-sensitized titania.^32,36,39^ To investigate the effect of the specific surface area, commercial titania (P90) that exhibits a specific surface area (94 m^2^ g^–1^) comparable to that of SGHT-200 was tested in the reductive cleavage of PPEn under blue light. We found that P90 showed activity similar to that of P25 (entries 3 and 4, Table 2). This suggests that the higher specific surface area of titania alone may not likely enhance the activity of ethanol-sensitized TiO_2_ for the reductive cleavage of PPEn. The role of the phase composition of titania was also assessed by carrying out a reaction using commercial anatase, commercial brookite, and a physical mixture of both catalysts. However, none of these three catalysts showed any activity (entries 5–7, Table 2), which signifies that some other features of titania play a larger role than the phase composition to achieve high activity in the reductive cleavage of PPEn. The surface OH groups of titania play a key role in surface-complex formation and can affect the activity of LMCT-sensitized titania,^32,36^ as they can serve as an adsorption site for ethanol. The relatively lower activity of commercial titania photocatalysts compared to SGHT-200 may relate to the smaller surface OH group density of P25 (8 OH nm^–2^) and P90 (5 OH nm^–2^)^50^ compared to titania synthesized via a hydrothermal method (10 OH nm^–2^).

**Table 2:**
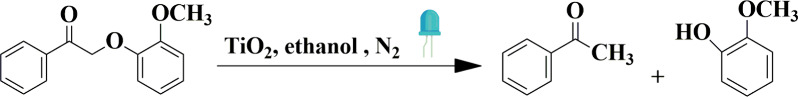
Photocatalytic Reductive Cleavage of PPEn in Ethanol under Blue Light (440 nm)^a^

entry	photocatalyst	additive	PPEn conversion (%)	acetophenone selectivity (%)	guaiacol selectivity (%)
1	none	none	3	0	0
2	SGHT-200	none	58	84	74
3	P25	none	20	80	92
4	P90	none	21	70	68
5	anatase	none	2	0	0
6	brookite	none	0	0	0
7	anatase:brookite	none	0	0	0
8	N-P25-U(1:3)	none	84	84	81
9	N-SGHT-U(1:3)	none	79	85	76
10	N-SGHT-U(1:1)	none	65	81	74
11	N-SGHT-U(1:5)	none	72	85	74
12	N-SGHT-IPA(1:3)	none	87	83	81
13	N-SGHT-NBA(1:3)	none	91	85	79
14	F-SGHT-200	none	0	0	0
15	F-N-SGHT-U(1:3)	none	0	0	0
16	SGHT-200-C-600	none	18	75	89
17	N-SGHT-U(1:3)-C-600	none	15	69	85
18	SGHT-200	AgNO_3_	0	0	0
19	N-SGHT-U(1:3)	AgNO_3_	0	0	0
20^b^	SGHT-200	none	5	0	0
21^b^	N-SGHT-U(1:3)	none	0	0	0
22	SGHT-200	DIPEA	54	90	86
23	N-SGHT-U(1:3)	DIPEA	84	76	68
24^c^	N-SGHT-U(1:3)	none	91	87	79

a Reaction conditions: 0.5 mM PPEn (0.0075 mmol) catalyst loading (3 g/L), solvent (ethanol), 0.5 mM PPEn solution volume (15 mL), AgNO_3_ (0.0075 mmol), N_2_ atmosphere, irradiation time (6 h).

b Experiment performed without N_2_ flow.

c Irradiation time (17 h); light source: Kessil PR160 L LED, 440 nm (max 44 W); average intensity: 399 mW/cm^2^ (measured from 1 cm distance).

Next, we tested nitrogen-doped titania photocatalysts synthesized using urea as a nitrogen dopant in the reductive cleavage of PPEn. N-P25-U(1:3) exhibited high PPEn conversion (84%) with high selectivity (>80%) for target fragmentation products under blue light (entry 8, Table 2). The noteworthy activity of N-P25-U(1:3) is ascribed to nitrogen doping, which enhanced the visible light absorption (Figure 3a) and reduced the band gap of P25 (Figure 3b). Additionally, the hydrothermal treatment of P25 during nitrogen doping can enhance the OH group density, which further improves the adsorption of ethanol and the activity of N-P25-U(1:3). Parrino et al. reported that the OH group density of P25 could be increased up to 11.9 OH nm^–2^ after hydrothermal treatment, which improves the degree of functionalization of TiO_2_ with silanes.^51^ The lab-synthesized nitrogen-doped photocatalyst, N-SGHT-U(1:3), also showed remarkable activity for PPEn conversion (79%), with high selectivity (>75%) for target fragmentation products (entry 9, Table 2). Moreover, we observed that the conversion of PPEn was slightly decreased by changing the molar ratio of Ti­(Oi-Pr)_4_ to urea from 1:3 to 1:1 (entries 9 and 10, Table 2). Furthermore, increasing the molar ratio of urea by 5 times did not result in any improvement in the activity of the catalyst (entry 11, Table 2). Additionally, we found that changing the molar ratio of urea did not affect the selectivity of the guaiacol and acetophenone (entries 9–11, Table 2), although we observed that increasing the molar ratio of the urea results in a relatively smaller crystal size and larger specific surface area (entries 2–4, Table 1), which could be beneficial in many catalytic reactions. However, in this study, we observed that the surface OH groups play more of a crucial role than the crystal size, phase composition, and specific surface area of titania. In addition, we observed that the nitrogen-doped titania photocatalysts prepared using different molar ratios of Ti­(O*i*-Pr)_4_ to urea exhibit similar absorption spectra (Figure S12) and band gaps (Figure S13), which is consistent with the comparable activity of these photocatalysts. Moreover, iso-propylamine (N-SGHT-IPA(1:3)) and *n*-butylamine (N-SGHT-NBA(1:3)) as nitrogen dopants further improved the activity of nitrogen-doped titania, reaching 87 and 91% PPEn conversion, respectively, without compromising the selectivity of acetophenone and guaiacol (entries 12 and 13, Table 2).

This indicates that the nitrogen doping significantly enhanced the activity of titania in the reductive cleavage of β-O-4 ketone under blue light (440 nm), which is likely related to improved visible light absorption. This is also indicated by the DRS UV–visible absorption spectra of nitrogen-doped titania samples that show that the light absorption has been enhanced in the 420–460 nm region after nitrogen doping (Figure 2a), with a marginal decrease in the band gap (Figure 2b). Moreover, nitrogen doping of titania often leads to the formation of oxygen vacancies, which can create midgap energy levels and tends to improve visible light absorption. However, there is no substantial difference observed in the absorption profile of the nitrogen-doped titania photocatalyst prepared using different nitrogen dopant sources (Figure 2a). N-SGHT-NBA(1:3) showed a relatively pronounced absorption in the visible region and consequently exhibited better conversion for PPEn. However, the selectivity for guaiacol and acetophenone is not affected by using different nitrogen dopants (entries 9–13, Table 2). It is presumed that for nitrogen-doped titania photocatalysts, both LMCT sensitization by ethanol and nitrogen doping likely enhance the visible light absorption and the activity of titania. We then tested the activities of SGHT-200 and N-SGHT-U(1:3) under slightly higher wavelength blue light (456 and 467 nm). Both catalysts showed moderate activity (entries 3 and 4, Table 3) for PPEn conversion and high selectivity for guaiacol and acetophenone under 456 nm light. However, SGHT-200 and N-SGHT-U(1:3) were found to be inactive for the reductive cleavage of β-O-4 ketone under 467 nm (entries 5 and 6, Table 3). This indicates that LMCT sensitization and nitrogen doping of titania induce activity in the near blue light spectrum (400–456 nm) due to a slight shift in the absorption in the blue region, which is also apparent from the DRS UV–visible results (Figure 2a).

**Table 3:**
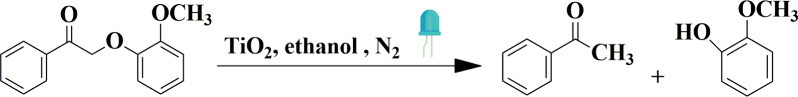
Effect of Irradiation Wavelength on the Photocatalytic Performance of Titania in the Reductive Cleavage of PPEn^a^

entry	photocatalyst	*h*ν (nm)	PPEn conversion (%)	acetophenone selectivity (%)	guaiacol selectivity (%)
1	SGHT-200	440	58	84	74
2	N-SGHT-U(1:3)	440	79	85	76
3	SGHT-200	456	41	85	80
4	N-SGHT-U(1:3)	456	39	81	79
5	SGHT-200	467	1	0	0
6	N-SGHT-U(1:3)	467	7	0	0

a Reaction conditions: 0.5 mM PPEn (0.0075 mmol) catalyst loading (3 g/L); solvent (ethanol); 0.5 mM PPEn solution volume (15 mL); N_2_ atmosphere; irradiation time (6 h); light source: 440 nm (max 44 W), 456 nm (max 50 W), 467 nm (max 44 W); average intensity: 399 mW/cm^2^ (measured from 1 cm distance).

Next, we investigated the effect of the solvent on the photocatalytic performance of SGHT-200 in the reductive cleavage of PPEn (Figure S14). We found that SGHT-200 exhibits a substantially high conversion of PPEn in ethanol (58%) compared to other aliphatic alcohols (methanol, 1-propanol, and 2-propanol). The high activity of SGHT-200 in ethanol may arise from the dissociative adsorption of ethanol on titania surface, which ensures better charge transfer.^52^ Studies have shown that the methanol adsorbs on the surface of titania preferably through molecular adsorption mode.^53–56^ The dissociative adsorption of methanol is favored at defect sites (oxygen vacancy site).^56,57^ The negligible PPEn conversion (3%) in the presence of methanol may be related to its molecular adsorption via intermolecular hydrogen bonding on the titania surface. Idriss and co-workers carried out charge density and work function analyses and found that the molecular adsorption mode results in a smaller charge transfer compared to the dissociative adsorption mode.^52^ This likely results in the negligible activity of SGHT-200 in the LMCT-mediated conversion of PPEn in methanol. Similarly, 1-propanol and 2-propanol are reported to follow preferably molecular adsorption mode on TiO_2,_ ^58^ which reduces the activity of SGHT-200 (<20% PPEn conversion) in the presence of these alcohols (Figure S14).

Furthermore, SGHT-200 and N-SGHT-U(1:3) have been evaluated to assess the scope of the reductive cleavage of β-O-4 ketones (Table 4). Both photocatalysts showed good selectivity (>70%) for β-O-4 ketone cleavage products. However, the conversion of β-O-4 ketones was significantly attenuated by the presence of electron-donating functional groups (methoxy groups) on the acetophenone ring (entries 5 and 6, Table 4). We hypothesized that this attenuation arises from the higher-energy LUMO of the donated ligand models, leading to a less favorable interaction between the substrate and Ti^3+^. Additionally, we tested the cleavage of β-O-4 alcohol (2-(2-methoxyphenoxy)-1-phenylethanol); however, this method was not successful for the cleavage of β-O-4 alcohol. This may be related to the competitive adsorption of ethanol and β-O-4 alcohols on titania.

**Table 4:**
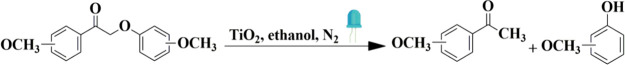

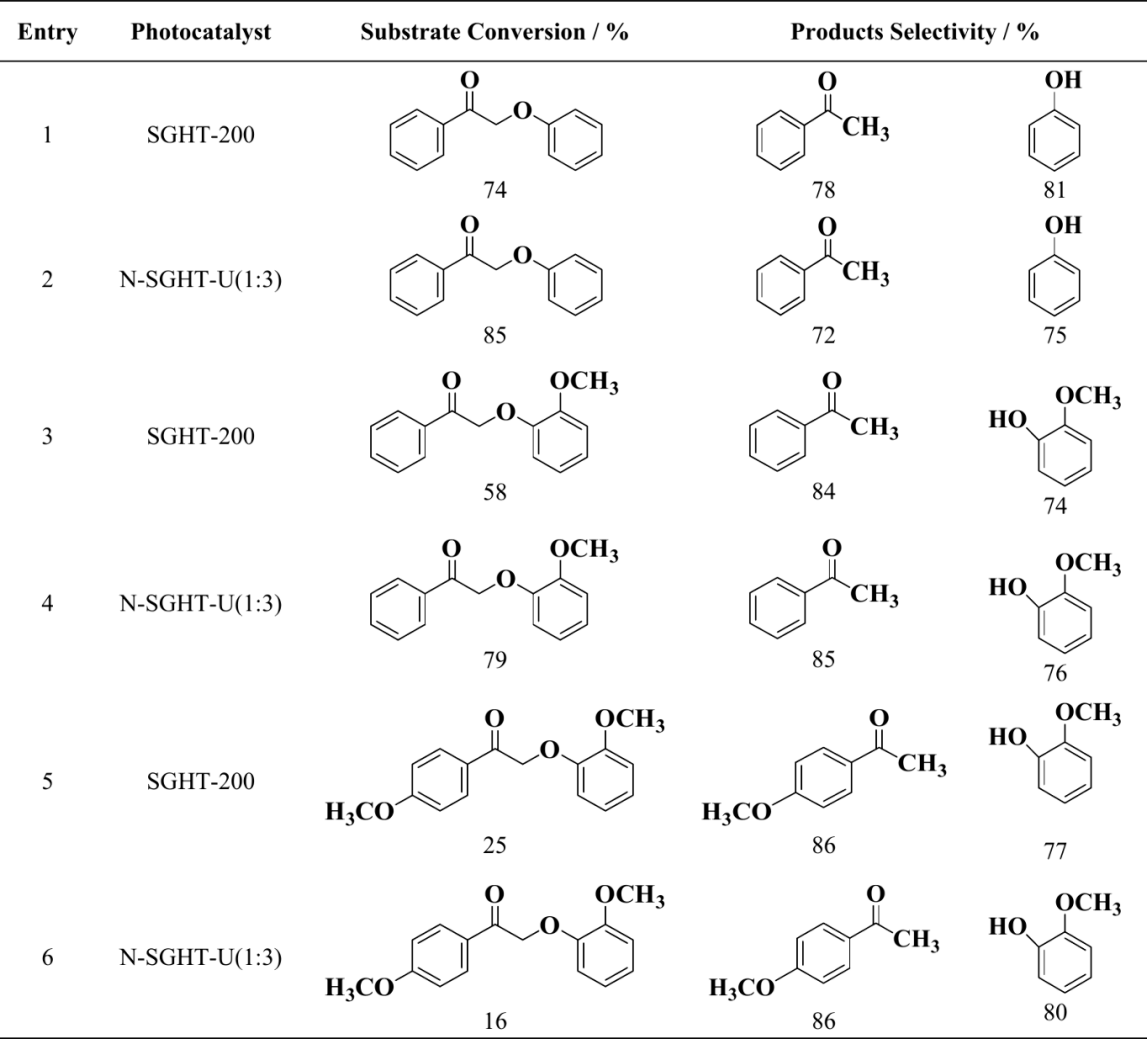
Photocatalytic Reductive Cleavage of β-O-4 Ketones in Ethanol under Blue Light^a^

a Reaction conditions: β-O-4 ketone (0.0075 mmol) catalyst loading (3 g/L, 45 mg); solvent (ethanol); 0.5 mM β-O-4 ketone solution volume (15 mL); N_2_ atmosphere; irradiation time (6 h); light source: Kessil PR160 L LED 440 nm (max 44 W).

The time course experiments performed (Figures S15–S20) for the reductive cleavage of β-O-4 model ketone show that the acetophenone selectivity remains high (>80%) over the period of time, while the selectivity of guaiacol slightly decreases with time. This could be related to the readsorption of guaiacol on the titania surface or slow desorption of guaiacol from the titania surface with time. The GC–MS analysis showed the formation of 2-(2-methoxyphenoxy)-1-phenylethanol (Figures S21 and S22) in addition to guaiacol and acetophenone. However, the selectivity observed for 2-(2-methoxyphenoxy)-1-phenylethanol was very low (12%) compared to those of guaiacol (70%) and acetophenone (85%). No other side products were observed based on the GC–MS analysis, which indicates that the slight decrease in guaiacol selectivity may not be related to the degradation of guaiacol. Other compounds detected in the GC–MS analysis (Figures S21 and S22) are related to column bleed and contamination from the septum.^39^ Moreover, a high mass balance (94%) was observed, which indicates that the proposed LMCT-assisted method effectively converts the lignin model compound to the desired cleavage product with minimal side reactions.

Next, we tested the activity of SGHT-200 in the photocatalytic cleavage of naturally derived ethanosolv lignin (a type of organosolv lignin). The GC–MS analysis showed the formation of acetic acid, 2-*tert*-butyl-4-methylphenol, 1-*tert*-butyl-4-phenoxybenzene, ethyl palmitate, and acetic acid-*n*-octadecyl ester after the photocatalytic cleavage of ethanosolv lignin (Figures S23 and S24). In a control reaction, we observed that the photolysis of ethanosolv lignin showed relatively similar products (Figure S25), which indicates that ethanosolv lignin undergoes some direct degradation under blue light. Additionally, diphenyl ether, butylated hydroxytoluene, and 2,2′-methylenebis­[4-methyl-6-*tert*-butylphenol] were detected by GC–MS analysis in both reactions (Figures S23 and S25). The last two compounds have been reported as lignin degradation products in the literature.^59,60^ We suspect that these are not related to lignin degradation in this study, but rather are formed as a result of contamination from the septum used to cover the photoreactor.^39^ We suggest that developing a photocatalytic method that works under lower-energy green light in future studies would help overcome the uncontrolled photolysis of lignin.

Natural lignin is usually found in a reduced form; therefore, we also performed the oxidation of ethanosolv lignin prior to reductive cleavage, following an oxidation protocol that successfully oxidized the β-O-4 model alcohol (TEMPO/NaOCl, Figure S26). Both unmodified ethanosolv lignin and oxidized ethanosolv lignin were characterized via IR analysis to evaluate the oxidation of ethanosolv lignin (Figure S27). We observed a clear shift in O–H stretching vibrations from 3400 cm^–1^ to a lower wavenumber (∼3140 cm^–1^) in oxidized lignin (Figure S27), which is related to the increased hydrogen bonding^61,62^ due to the introduction of more carbonyl-containing functional groups such as carboxylic acids, ketones, and potential aldehyde groups. The band at 1725 cm^–1^, which is generally attributed to nonconjugated carbonyl group (C=O) stretching, is relatively more pronounced after oxidation; this suggests the oxidation of alcohol and generation of new carbonyl groups or carboxyl groups.^63,64^ However, we did not see clear evidence of the formation of conjugated carbonyl groups (1680–1700 cm^–1^) by the oxidation of desired benzylic alcohols. Additionally, the intensities for C–O stretching bands in the 1025–1230 cm^–1^ region are reduced in oxidized lignin (Figure S27), which may be related to the conversion of C–O single bonds to carbonyls. Unfortunately, the reductive cleavage of oxidized ethanosolv lignin did not show any improvement in terms of the formation of target products (guaiacol and acetophenone). We noted a few aromatic products, butylated hydroxytoluene, 5-isopropyl-2-methylphenyl heptanoate, and 2,2′-methylenebis­[4-methyl-6-*tert*-butylphenol], in the cleavage of oxidized ethanosolv lignin (Figure S28).

The complex nature and diverse functional groups of the as-obtained ethanosolv lignin and oxidized ethanosolv lignin make the selective conversion of lignin challenging. The OH groups present in lignin may interact with the surface OH groups of titania and interfere with the photocatalytic activity of titania in ethanosolv lignin conversion. Although the lignin itself does not absorb visible light (Figure S29), the DRS UV–visible absorption spectrum of titania exposed to ethanosolv lignin solution shows a distinct red shift (Figure S30), which suggests the interaction of ethanosolv lignin with titania. Similarly, SGHT-200 exposed to guaiacol solution also showed a pronounced increase in visible light absorption (Figure S31), which indicates that phenolic OH groups in natural lignin may interact with titania and affect its photocatalytic activity. We propose that the acetylation of natural lignin to replace the hydroxyl groups of lignin with acetyl groups would be helpful to overcome this challenge in future studies. Moreover, it will also improve the solubility of natural lignin in organic solvents, which is crucial for the photocatalytic valorization of natural lignin.

Furthermore, we found that SGHT-200 has not been able to maintain its activity in the recyclability test, and 3% conversion for PPEn was observed in the second run (Figure S32) and no activity was observed in the third and fourth runs. Similarly, N-SGHT-U(1:3) did not show any activity in the reusability test. Our previous work showed that the adsorption of guaiacol and degradation products of the sensitizer (triethylammonium tetraphenyl borate, TEAH BPh_4_) in the LMCT-mediated reductive cleavage of lignin model compounds can lead to the reduced activity of titania in multiple runs.^39^ To derive further insights into the reduced activity of titania in the reusability test, we carried out a detailed characterization of reused SGHT-200. We observed that the crystal size and phase composition of SGHT-200 did not change significantly after multiple cycles (Figure S33 and Table S1). However, the specific surface area of SGHT-200 is slightly reduced after reuse (Table S1), which may be related to the adsorption of guaiacol or initially adsorbed ethanol. Sun and co-workers reported that the carbon accumulation on the surface of TiO_2_ during organic wastewater treatment leads to a slight reduction in the surface area of titania.^65^ Moreover, the red shift in the DRS UV–visible absorption spectrum of guaiacol-adsorbed titania (Figure S31) suggests that the guaiacol produced by the reductive cleavage of PPEn may adsorb over the surface of titania, which offers a competition for the adsorption of ethanol and interferes with its activity in the recyclability test. Next, we recorded the DRS UV–visible absorption spectra of reused catalysts. It can be seen in Figure S34 that the absorption spectra of reused titania catalysts showed a marked increase in visible light absorption. This red shift is induced possibly due to the adsorption of guaiacol and trace amount of β-O-4 alcohol, which do not desorb from the surface of titania after washing with acetonitrile and water and may result in the deactivation of the catalyst in the next run. Furthermore, IR analysis of reused SGHT-200 (Figures S35 and S36) exhibits weak bands at 1444 and 1549 cm^–1^, which may be related to the adsorption of guaiacol and initially adsorbed ethanol. Moreover, the O–H stretching vibration associated with the surface OH groups of titania was not observed even after washing with water (Figure S36). These results are consistent with our previous studies carried out on TEAH BPh_4_-sensitized titania in the reductive cleavage of β-O-4 ketone.^39^ This suggests that even after washing with solvents, some amount of guaiacol may remain adsorbed over the surface of the catalyst, which is also indicated by the yellow color of reused SGHT-200 (Figure S37). This inhibits the adsorption of ethanol in the next cycle and makes the reuse of SGHT-200 challenging in reductive cleavage of β-O-4 ketone.

The turnover number (TON) is a key metric to evaluate the efficiency of photocatalytic systems. Cossairt and co-workers found that CdSe quantum dots showed a higher TON (22 × 10^3^, estimated from figure) than an iridium-based catalyst (7 × 10^3^, estimated from the figure) at 0.003 mol % catalyst loading in the reductive cleavage of β-O-4 ketone at optimized conditions.^21^ It is important to consider that in heterogeneous photocatalysis, reporting the activity in terms of TON is less appropriate, because even though there is a complete theoretical estimation of the number of active sites, the number of photocatalytically active sites is still unknown. Some of the potentially active sites are not irradiated due to the aggregation of catalysts and shading, and they do not take part in catalysis.^66^ However, the TON cannot be completely ruled out in heterogeneous photocatalysis, as it provides a rough estimation of the activity of the catalyst based on potential active sites. The surface hydroxyl groups of titania are considered as potential adsorption sites and active sites in this study. We evaluated the photocatalytic activity of titania based on surface hydroxyl groups. The low conversion of β-O-4 ketone per surface OH groups by SGHT-200 and P25 (entries 1 and 2, Table 5) indicates that all of the hydroxyl groups are not necessarily accessible to ethanol for adsorption to activate the catalytic cycle. In addition, all surface areas are not accessed by light, especially inside the pores and aggregated particles. Our results are consistent with the previous studies carried out on the LMCT-mediated oxidation of 5-hydroxymethylfurfural (HMF) by sol–gel and hydrothermal titania, which also shows the low conversion of HMF (0.08) per surface OH groups.^36^

**Table 5:** Photocatalytic Performance of Titania per Surface OH Groups

entry	catalyst	OH group density/nm^2^	moles of OH groups	PPEn conversion per OH groups
1	SGHT-200	10	8.14 × 10^–5^	0.05
2	P25	8	2.74 × 10^–5^	0.05

Furthermore, we performed actinometric experiments (Figure S38), and the quantum yields (Φ) observed for guaiacol and acetophenone in the reductive cleavage of β-O-4 ketone by N-SGHT-U(1:3) were 2 and 3%, respectively, under blue light (440 nm). Similarly, SGHT-200 showed 2% quantum yields for guaiacol and acetophenone. The low quantum yields suggest that the reaction does not follow a radical chain mechanism. Our previous work on the reductive cleavage of β-O-4 ketone by triethylamine-sensitized titania showed comparable quantum yields (3–4%).^39^ Similarly, past studies carried out on the LMCT-mediated oxidation of 5-hydroxymethylfurfural and benzyl alcohol by TiO_2_ under visible light showed 6 ^36^ and 5%^32^ quantum yields, respectively. The low quantum yields in heterogeneous photocatalytic reactions in the batch photoreactor are primarily related to the scattering and reflection of light by solid suspended photocatalysts, which reduces the number of photons available to drive the LMCT process. The quantum yield in heterogeneous photocatalysis can be improved by designing photoreactors that minimize scattering and reflection and maximize light penetration. The flow photoreactors can help overcome this challenge in future studies by ensuring improved light utilization and mass transfer due to a high surface area to volume ratio, which can enhance the overall efficiency of photon utilization.

### LMCT Complex Formation

We hypothesized that the use of ethanol as a solvent in the reaction may lead to ethanol adsorption on the surface of titania and form a surface complex that is active under blue light. To investigate this proposition, DRS UV–visible absorption spectra were recorded for undoped titania samples and ethanol-adsorbed titania. A marginal red shift was observed in the absorption spectra of SGHT-200 after ethanol adsorption (Figure 5a). Electron-rich adsorbates such as aryl alcohols tend to induce absorption up to the far-visible light region of the spectrum.^30,67^ The adsorption of a relatively less electron-rich alcohol, ethanol, induces a less intense red shift in the absorption spectrum of titania; however, it enables the reductive cleavage of β-O-4 ketone under blue light. Scaiano and co-workers also observed a marginal shift in the absorption when indole was employed as a sensitizer for titania.^37^ Interestingly, the indole-sensitized titania efficiently catalyzed the Diels–Alder reaction between indole and 1,3-cyclohexadiene under blue light.^37^ Similarly, Higashimoto et al. found that benzyl alcohol can form an LMCT complex on the surface of titania and slightly shifts the absorption into the blue region of the spectrum, which enabled the oxidation of benzyl alcohol to benzaldehyde under blue light.^68^ These studies demonstrate that the slight red shift in the absorption spectrum of titania may induce activity under blue light.

**Figure 5:**
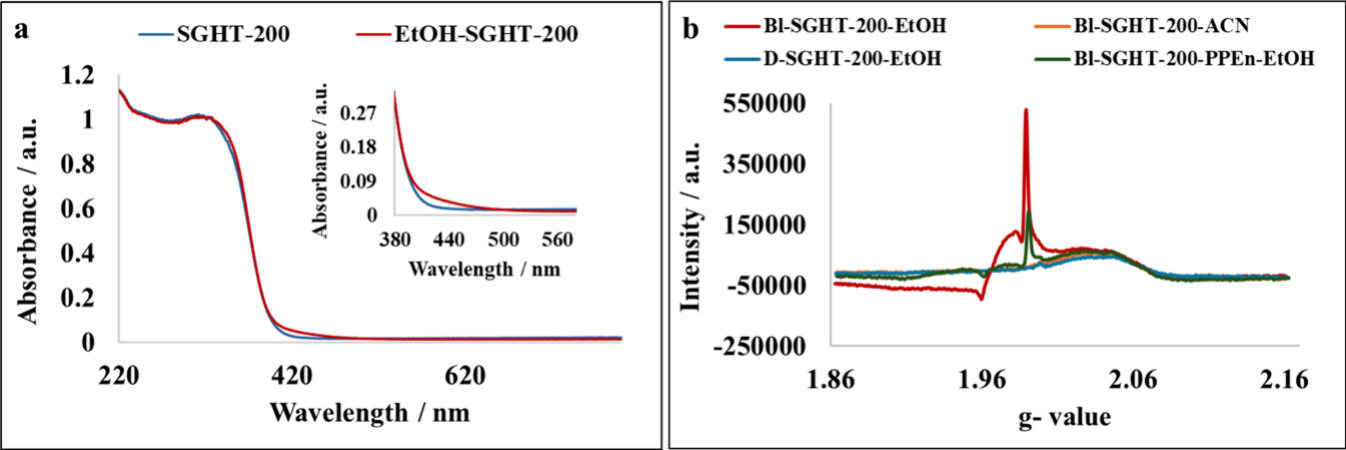
(a) DRS UV–visible absorption spectra of SGHT-200 and ethanol-adsorbed titania (EtOH-SGHT-200). (b) EPR spectra of blue light (440 nm)-irradiated titania suspension in ethanol and acetonitrile.

The adsorption of ethanol on titania has been extensively studied by researchers,^52,69–71^ which provides valuable insights into the LMCT complex formation on titania by the adsorption of ethanol. Erdőhelyi and co-workers reported that ethanol can adsorb on the surface of titania molecularly and dissociatively.^70^ In molecular adsorption, ethanol is weakly adsorbed on the surface of titania through hydrogen bonding with the surface OH groups of titania. However, in dissociative adsorption, ethanol undergoes deprotonation to form ethoxy species, which coordinate to the Ti site; this is accompanied by the evolution of water molecules. DFT studies showed that the dissociative adsorption of ethanol is relatively more favorable due to the stable configuration, in contrast to molecular adsorption.^52,69^ The hydroxylated titania surface further favors the dissociative adsorption of ethanol over molecular adsorption.^72^ Furthermore, charge density and work function analyses performed by Idriss and co-workers showed that the dissociative adsorption results in larger charge transfer from ethanol to titania compared to molecular adsorption.^52^ This is very beneficial for the LMCT-mediated activity of titania.

To further investigate the adsorption of ethanol on titania and subsequent complex formation, the ethanol-adsorbed titania sample was studied by IR analysis. We found that the intensity of the band related to the O–H stretching vibration in SGHT-200 was reduced after ethanol adsorption (Figure 6). This indicates that surface OH groups of titania act as adsorption sites for ethanol molecules, and ethanol undergoes dissociative adsorption on titania. Moreover, the band appearing at 1405 cm^–1^ corresponds to the bending vibrations of the methyl group. Erdőhelyi and co-workers carried out a detailed IR study on the gas phase adsorption of ethanol on TiO_2_.^70^ It was found that the bands related to the O–H stretching vibrations disappeared after the adsorption of ethanol, which indicates that the OH groups of titania are involved in the adsorption of ethanol. Ethanol likely undergoes deprotonation to form an ethoxy species, which coordinates to the Ti site. Additionally, bands related to the C–H stretching vibration (2969–2867 cm^–1^) and C–O stretching vibrations (1065–1050 cm^–1^) were observed, which are attributed to the adsorption of ethanol.^70^

**Figure 6:**
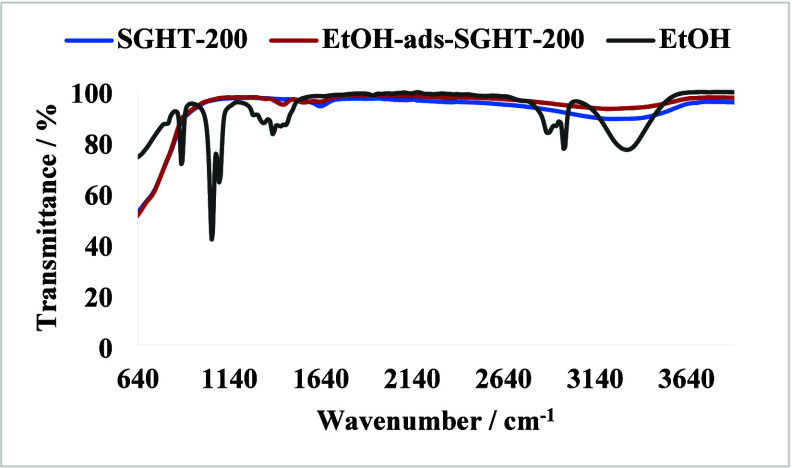
IR spectra of titania and the ethanol-adsorbed titania sample.

We hypothesized that the surface hydroxyl groups play a crucial role in the adsorption of ethanol and the visible light activity of titania in β-O-4 ketone cleavage. To corroborate this hypothesis, the activities of surface-fluorinated titania and calcined titania were assessed in β-O-4 ketone (PPEn) cleavage under blue light (440 nm). Fluorinated titania photocatalysts were inactive for the reductive cleavage of ketone under blue light (entries 14 and 15, Table 2), whereas the calcined titania photocatalysts showed a significant decline in activity (entries 16 and 17, Table 2). This provides evidence that the surface OH groups on titania are essential for the adsorption of ethanol and formation of visible light active surface complexes for the reductive cleavage of β-O-4 ketone (PPEn).

To further confirm that the surface complexation of ethanol results in the in situ generation of Ti^3+^ under blue light, we decided to use electron paramagnetic resonance (EPR) analysis to detect the presence of Ti^3+^ in the reaction medium. The nonirradiated titania (D-SGHT-200-EtOH) suspension in ethanol did not show any Ti^3+^ signal (Figure 5b). However, the irradiated sample showed signals at *g* = 1.96 and 1.98 (Figure 5b) related to Ti^3+^.^73,74^ The formation of Ti^3+^ is also evidenced by the change in color of titania from white to bluish gray (Figure S39). The irradiated N-SGHT-U(1:3) photocatalyst also showed similar signals under blue light irradiation in ethanol (Figure S40). Moreover, when the titania suspension was irradiated in acetonitrile as a solvent, no Ti^3+^ signal was detected (Figure 5b). We therefore concluded that Ti^3+^ ions were formed in situ by accepting an electron generated under blue light irradiation mediated by the ethanol surface complex.

### Mechanistic Studies

To gain further insights into the role of Ti^3+^ in the cleavage of β-O-4 ketone, the reductive cleavage of PPEn was carried out in the presence of the electron acceptor [silver­(I) nitrate]. The addition of silver­(I) cations as an acceptor of electrons completely stopped the photocatalytic activity of N-SGHT-U(1:3) and SGHT-200 (entries 18 and 19, Table 2). This suggests that the oxidation of Ti^3+^ by silver­(I) inhibits the reduction of the β-O-4 ketone. Moreover, when the reaction was performed without nitrogen flow, SGHT-200 showed negligible conversion for PPEn (entry 20, Table 2) and N-SGHT-U(1:3) did not show any activity (entry 21, Table 2). This indicates that the Ti^3+^ generated is rapidly oxidized to Ti^4+^ by the naturally dissolved oxygen in the reaction medium and thus is unable to take part in the reductive cleavage of PPEn. The use of *N*,*N*-diisopropylethylamine (DIPEA) as a hole scavenger did not significantly affect the PPEn conversion (entries 22 and 23, Table 2). This suggests that holes may not be formed, as the activity of titania is attributed to LMCT sensitization by ethanol under blue light.

Based on the mechanistic experiments, a plausible reaction mechanism for the reductive cleavage of β-O-4 ketones is presented in Figure 7. Ethanol is deprotonated and forms a surface complex on the surface of titania. The blue light (440 nm) irradiation of this surface complex transfers an electron from the ethoxide into the conduction band of titania, while reducing Ti^4+^ to Ti^3+^. The in situ-generated Ti^3+^ reduces the β-O-4 ketone to the radical anion, which undergoes a β-scission to form a phenoxy radical and the acetophenone enolate. A hydrogen atom transfer event between ethanol and the phenoxy radical intermediate could form guaiacol, while the protonation of the intermediate enolate by ethanol could generate acetophenone.

**Figure 7:**
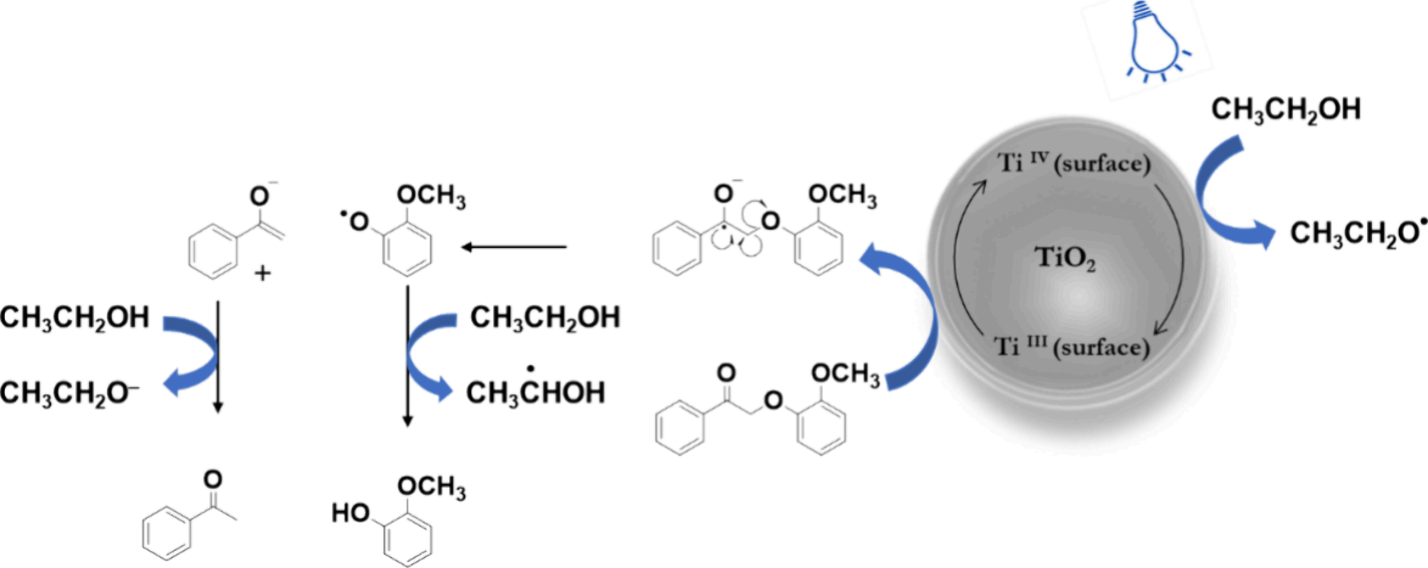
Plausible reaction mechanism of reductive cleavage of the β-O-4 ketone in ethanol under blue light (440 nm).

Our work overcomes significant challenges related to the development of sustainable methods for the selective cleavage of β-O-4 lignin model compounds. For instance, previous studies reported the reductive cleavage of the C–O bonds of β-O-4 ketones in the presence of sodium acetate using TiO_2_ ^24^ and NiO/TiO_2_ ^22^ as catalysts under UV light (365 nm). We demonstrated that the LMCT sensitization of titania by ethanol and nitrogen doping of titania helped achieve the β-O-4 ketone cleavage without the use of an additive under relatively mild blue light with similar selectivity for target fragmentation products. Additionally, prior studies require precious iridium-based catalysts^19^ or toxic Cd-based catalysts (CdSe quantum dots)^21^ to accomplish β-O-4 lignin model ketone conversion under blue light and white light, respectively. Our work provides a sustainable solution to this problem by employing an inexpensive and relatively nontoxic TiO_2_ catalyst to realize the reductive cleavage of β-O-4 ketones under blue light through an LMCT-assisted approach. This study demonstrates that the LMCT sensitization of titania by ethanol provides an additive-free and mild strategy for the reductive cleavage of β-O-4 lignin model ketones without an additional reductant. We expect that the visible-light-driven LMCT-mediated approach reported here will unlock potential in related organic transformation and biomass valorization, allowing reduction under more selective and sustainable conditions. However, more efforts are required to improve the performance of the LMCT-mediated photocatalytic method for a wide range of substrates and natural lignin molecules.

## Conclusions

Pristine titania is intrinsically unable to absorb visible light due to its wide band gap and is therefore catalytically inactive under visible light. Herein, we develop an LMCT-mediated photocatalytic system consisting of titania sensitized by ethanol for the reductive cleavage of β-O-4 ketones into valuable aromatic compounds under blue light. Light irradiation initiates the electron transfer from ethanol to the conduction band of titania and generates Ti^3+^_,_ which catalyzes the reductive cleavage of the β-O-4 bond in lignin model ketones. EPR analysis provides evidence that the LMCT sensitization of titania by ethanol generates Ti^3+^ upon blue light irradiation. Furthermore, DRS UV–visible analysis shows that the nitrogen doping of titania slightly enhances the visible light absorption of titania up to 500 nm, which further improves the activity of titania in the reductive cleavage of β-O-4 ketones. The experiments carried out by fluorinated titania and calcined titania show that the surface hydroxyl groups of titania are crucial for the LMCT sensitization and reductive cleavage of β-O-4 ketones under visible light. Moreover, achieving high conversion of β-O-4 ketone with electron-donating functional groups on the acetophenone ring remains a challenge to be addressed. Overall, our results offer a promising direction for the development of mild and sustainable visible-light-driven titania-based photocatalytic systems for the organic transformation through the LMCT-mediated process.

## Supplementary Material



## Notes

Detailed synthesis and experimental procedures, supplementary figures, catalyst characterizations, and ^1^H NMR spectra for all substrates (PDF)
