# Supplementary material for: Additive-Free Reductive Cleavage of Lignin Model Compounds by Ethanol-Sensitized Titania under Visible Light: A Sustainable Approach to β‑O‑4 Bond Cleavage
Source: ACS Sustain Chem Eng. 2025 Nov 6;13(45):19482–95. doi: 10.1021/acssuschemeng.5c03068 (PMC12628334; doi:10.1021/acssuschemeng.5c03068)
Supplement: Supplementary file 1 [file sc5c03068_si_001.pdf]

Supporting Information

Additive Free Reductive Cleavage of Lignin Model Compounds by Ethanol-Sensitized Titania Under Visible Light: A Sustainable Approach to β-O-4 Bond Cleavage

Ayesha Khan†*, Logan W. Evans† and David B.C. Martin†*

†Department of Chemistry, University of Iowa, Iowa City, Iowa 52242, United States

*Corresponding authors' email: david-martin@uiowa.edu

ayesha-khan-1@uiowa.edu

Supporting Information Content:

Number of pages: 38

Number of Figures: 48

Number of Tables: 1

Chemicals: Titanium(IV) isopropoxide (97+%, Sigma Aldrich), 2-propanol (Sigma Aldrich 99.9%), commercial titania, P25 (99.5 %, Evonik), Urea (>98%, Sigma Aldrich), acetonitrile (99.9 %, Fisher Chemical), methanol (99.9 %, Fisher Chemical), silver nitrate (99.8 %), Potassium trioxalatoferrate(III) trihydrate (Thermo Scientific Chemicals), 1,10-Phenanthroline (99%, Alfa Aesar), anatase (99.7%, Thermo Scientific Chemicals) and brookite (99.9%, Sigma Aldrich) sodium fluoride (99 %, Acros Organics), Water (HPLC grade, Fisher Chemical), ethanol (>99.5%, HPLC grade, Sigma Aldrich)

Synthesis of undoped titania nanoparticles (SGHT-200)

In a typical procedure, 0.129 moles of titanium(IV) isopropoxide ($Ti(O\textit{i}\text{-}Pr)_4$) were dissolved in 25 ml of 2-propanol and vigorously stirred for 2 hours at room temperature. Next, 1 mL of 1 M nitric acid was added to the solution under continuous stirring, until a white gel is formed. Then, 25 ml of deionized water was added to the gel and stirred for 3 hours. The obtained precipitates were filtered, washed with deionized water multiple times, and dried at 110 °C in an oven for 24 hours. The dried nanoparticles were shifted to Teflon-lined stainless-steel autoclave filled with deionized water (70 mL) for hydrothermal treatment for 24 hours at 200 °C in an oven. The as-synthesized sample was named SGHT-200.

Preparation of fluorinated titania (F-SGHT-200)

The surface fluorination of SGHT-200 has been performed by adding 150 mg of SGHT-200 in 15 ml of 0.053 M NaF solution. The pH of the NaF solution was adjusted to 3 using 2 M HCL. The suspension was stirred in dark for 5 hours in dark to achieve surface fluorination. The fluorinated SGHT-200 (F-SGHT-200) was washed with water, filtered and dried at 110 °C for 24 hours. XPS measurement was performed to confirm the substitution of hydroxyl groups with fluorine (Figure S45 and S46)

Preparation of calcined titania (SGHT-200-C-600)

The calcination of SGHT-200 was performed in muffle furnace by heating the titania at 600 °C for 3 hours under static air at a heating rate of 5 °C / min.

Preparation of ethanol-adsorbed titania samples

For DRS UV-visible measurements and IR analysis, ethanol-adsorbed-SGHT-200 sample was prepared by suspending 150 mg of SGHT-200 in 15 mL of ethanol. The suspension was stirred for 1 h in dark at 400 rpm. The catalyst was then collected, dried at 80 °C for 12 hours.

Preparation of guaiacol-adsorbed SGHT-200

For DRS UV-visible measurements, guaiacol-adsorbed SGHT-200 (Guaiacol-SGHT-200) was prepared by adding 100 mg of SGHT-200 in 0.5 mM guaiacol solution (15 mL) in acetonitrile in the scintillation vial. The suspension was stirred for 1 hour in dark at 400 rpm. Afterwards, the catalyst was collected, dried at 80°C for 12 hours.

Preparation of ethanosolv lignin-adsorbed SGHT-200 (Lignin-SGHT-200)

2.0 mg of ethanosolv lignin was dissolved in 15 mL of ethanol and then filtered. Next, the ethanosolv lignin solution and 100 mg of SGHT-200 were loaded into a glass vial and stirred for 1 hour in dark at 400 rpm. Afterwards, the catalyst was collected, dried at 80°C for 12 hours.

Oxidation of β-O-4 lignin model alcohol and ethanosolv lignin

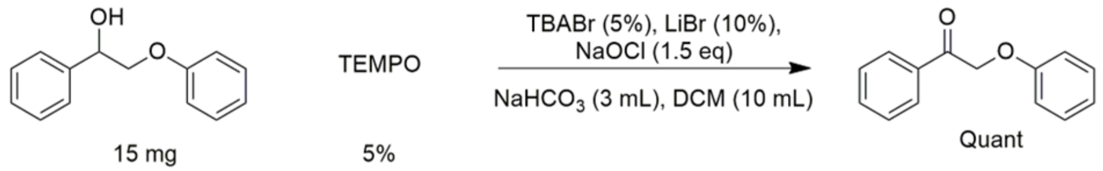

The lignin model compound was added to a 20 mL scintillation vial equipped with a stir bar. Then TBABr, LiBr, and TEMPO were added to the vial. 10 mL of dichloromethane were added and stirring began. 1.5 eq of NaOCl were added to the vial forming a biphasic mixture. The NaOCl was acidified by dropwise addition of $NaHCO_3$. The mixture was stirred for 10 minutes. The biphasic mixture was then separated via a separatory funnel and the aqueous layer was extracted with dichloromethane. The crude oxidation reactions on the model lignin compound were analyzed via NMR. The oxidized product was obtained in quantitative conversion (Figure S28). The same procedure was followed for the oxidation of ethanosolv lignin, except the ethanosolv lignin was allowed to stir in the dichloromethane for 4 hours before adding the other reagents.

Photocatalytic cleavage of ethanosolv lignin

Briefly, 2.0 mg of ethanosolv lignin was dissolved in 15 mL of ethanol and then filtered. Next, the ethanosolv lignin solution and 45 mg of SGHT-200 were loaded into a glass vial. The photoreactor was covered with the septum (14 x 20, VWR), sealed with the parafilm and placed into the Hepato Chem, EvoluChemTM PhotoRedOx Box (Figure S1) and stirred for 1 hour in the dark to establish an equilibrium. The suspension was then continuously bubbled with N_2 under blue light (Kessil PR-160L 440 nm) for 24 hours. The aliquots were collected and filtered using 0.22 μm nylon syringe filters and analyzed on GC-MS (Agilent 7250 GC/Q-TOF). The photocatalytic cleavage of oxidized ethanosolv lignin was carried out following the same method. The photolysis of ethanosolv lignin was performed in a similar way, except that the experiment was carried out without the photocatalyst.

Synthesis of β-O-4 lignin model compounds

All reactions were carried using oven dried or flame dried glassware charged with a magnetic stir bar and conducted under an inert nitrogen atmosphere using typical Schlenk techniques, unless otherwise noted. All solvents were dried by passage through columns of activated alumina or distilled and stored under nitrogen over freshly activated 4 Å sieves or otherwise freshly distilled. All starting materials were prepared according to known literature procedures or used as obtained

from commercial sources, unless otherwise indicated. Reactions were monitored by thin-layer chromatography (TLC) and carried out on 0.25 mm coated commercial silica gel plates (Analtech TLC Uniplates, F254 precoated glass plates) using UV light as visualizing agent. Unless otherwise indicated, silica gel chromatography was performed using flash chromatography on P60 silica.

Synthesis of 2-(2-methoxyphenoxy)-1-phenylethanone (PPEn)

2-(2-methoxyphenoxy)-1-phenylethanone was prepared according to literature procedures.[1] To a 250-mL round bottom charged with a stir bar, guaiacol (17.88 mmol, 1.25 eq) and potassium carbonate (21.46 mmol, 1.5 eq) was added acetone (60.0 mL, 0.24 M) and 2-bromoacetophenone (14.30 mmol, 1 eq) with N_2 atmosphere protection. The reaction was refluxed for 4 hours, followed by filtration through celite and then concentrated *in vacuo*. Following filtration, the crude product was recrystallized from ethanol to afford the product, 2-(2-methoxyphenoxy)-1-phenylethanone as a white solid in 80% yield.

Other phenylethanone substrates were prepared via the same preparation procedures, substituting different starting materials.

2-(2-methoxyphenoxy)-1-phenylethanone

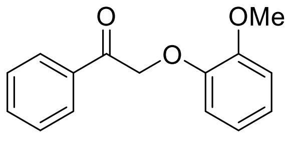

Spectral data matched literature sources.[2] ^{1}H NMR (500 MHz, $CDCl_3$) δ = 8.04-7.99 (m, 2H), 7.61 (t, *J* = 7.4 Hz, 1H), 7.49 (t, *J* = 7.4 Hz, 2H), 7.00-6.83, (m, 4H), 5.34 (s, 2H), 3.88 (s, 3H)

2-phenoxy-1-phenylethanone

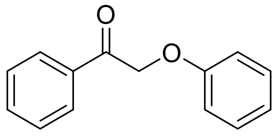

Spectral data matched literature sources.[2] Prepared from 2-bromoacetophenone and phenol in 83% yield. ^{1}H NMR (500 MHz, $CDCl_3$) δ = 8.01 (d, *J*= 7.4 Hz, 2H), 7.62 (t, *J*=7.4 Hz, 1H), 7.50 (t, *J*=7.7 Hz, 2H), 7.29 (t, *J*=8.0 Hz, 2H), 7.01-6.93 (m, 3H), 5.27 (s, 2H)

2-(2-methoxyphenoxy)-1-(4-methoxyphenyl)ethanone

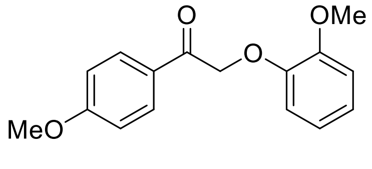

Spectral data matched literature sources.[2] Prepared from 2-bromo-4'-methoxyacetophenone and guaiacol in 82% yield. ^{1}H NMR (400 MHz, $CDCl_3$) δ = 8.02 (dd, *J*=6.9, 2.1 Hz, 2H), 6.99-6.89 (m, 4H), 6.86-6.82 (m, 2H), 5.28 (s, 2H), 3.89 (s, 3H), 3.88 (s, 3H)

Quantum yield measurement

The quantum yield (Φ) is defined as the ratio of moles of product produced to the number of moles of photon absorbed by the system per unit time (Equation 1).

$$\Phi = \frac{Number\ of\ moles\ of\ product\ produced\ per\ unit\ time}{Number\ of\ moles\ of\ photons\ (Einstein)\ absorbed\ per\ unit\ time} \times 100 \qquad (1)$$

To measure the quantum yield (Φ), the photon flux to the photoreactor was determined by potassium ferrioxalate actinometry.[3,4] The actinometry experiments were carried out in a dark room under blue light (440 nm). In a typical procedure, 15 mL of 0.15 M potassium ferrioxalate solution was charged into a glass scintillation vial used for photocatalytic experiments. Then the actinometer solution was illuminated for 60 s while stirring (400 rpm). At the same time, another sample prepared following the same procedure was left in the dark as a control. Then, an aliquot (3 mL) of 0.15 M potassium ferrioxalate was taken in glass vials and then 0.1% buffered 1,10-phenanthroline solution (500 μL) was added to irradiated and non-irradiated samples. The samples were then allowed to develop for half an hour in the dark. After that, the absorption of each of the sample was recorded Agilent Cary 5000 UV/vis/NIR spectrometer at 510 nm. The amount of Fe^{2+} formed during irradiation has been measured using the optical difference (ΔA 510 nm) between the irradiated and non-irradiated sample and the extinction coefficient at 510 nm (ε = 11,100 $M^{-1}cm^{-1}$)

$$Nhv = \frac{moles\ of\ Fe^{2+}}{\emptyset \times t \times F} \qquad (2)$$

$$F = 1 - 10^{-A} \qquad (3)$$

The quantum yield for Fe^{2+} production is known, the photon flux absorbed by the sample per unit time is estimated using Equation 2. Where t is the irradiation time in seconds and F is the fraction of light absorbed determined by using Equation 3.[5]

The photon flux of the photocatalytic set-up was calculated to be 7.85 × 10^{-9} einsteins s^{-1}using PR160L Kessil 440 nm lamp.

OH group density measurement:

The OH group density of SGHT-200 and P25 was determined via a thermogravimetric analysis (TGA) weight loss (Figure S45), performed using SDT-Q600. The method employed for TGA analysis involved two steps. In step 1, the sample was heated under air (40 mL min^{-1}) from 25 to 120 °C at 5 °C min^{-1} and held at this temperature for 3 h to remove physically bound water from the surface. In step 2, the temperature was increased to 750 °C at 10 °C min^{-1} and held for 1 h. The weight loss during step 2 was used to calculate the number of hydroxyl groups per surface area according to the following formula:

$$ {OH}/{nm^2} = \alpha \frac{2(W)\frac{N_A}{MH_2O}}{10^{18} \times SSA} \quad (4) $$

where W is the weight loss per mg of sample, SSA is the specific surface area (m^2/g), MW is the molecular weight of water (18.01 g/mol), N_A is Avogadro's constant (6.023×10^{23}), and α is the calibration coefficient (0.625).

2-(2-methoxyphenoxy)-1-phenylethanone (PPEn) conversion per surface OH group

The number of OH groups and conversion of PPEn with respect to active sites of SGHT-200 were calculated as follows

$$ \text{Number of OH groups} = D_{OH} \text{ x SSA x } m_{cat} \quad (5) $$

$$ moles\ of\ OH\ groups = \frac{\text{Number of OH groups}}{\text{NA}} \quad (6) $$

D_{OH} is the density of OH groups per m^2, N_A Avogadro's number, SSA (specific surface area of the catalyst in m^2g^{-1}), m_{cat} mass of catalyst in g.

$$ Conversion\ of\ PPEn\ per\ surface\ OH\ groups = \frac{\text{moles of PPEn converted after 6 hours}}{\text{moles of OH groups}} \quad (7) $$

Mass balance measurement:

The mass (M) balance was determined using following formula.

$$ \boldsymbol{Mass\ balance} = \frac{\mathbf{M}_{\boldsymbol{\beta-O-4}\ \mathbf{alcohol}} + \boldsymbol{M}_{\boldsymbol{guaiacol}} + \boldsymbol{M}_{\boldsymbol{acetophenone}} + \boldsymbol{M}_{\boldsymbol{unreacted\ \beta-O-4\ ketone}}}{\boldsymbol{M}_{\mathbf{initial}\ \boldsymbol{\beta-O-4}\ \mathbf{ketone}}} \times \mathbf{100} \quad (8) $$

Characterization

X-ray diffraction analysis of photocatalysts was performed on a Bruker D8 Advance Powder Diffractometer with an LYNXEYE detector. Nickel filtered Cu K-alpha radiation was used with a tube voltage of 40 kV and amperage of 40 mA. The data collection was over a 2-theta range of 5-90 degrees, with a step size of 0.02 degrees, and a collection time of 0.4 sec/step. The UV/visible diffuse reflectance spectroscopy (DRS) measurements of undoped and nitrogen-doped titania samples was carried out on an Agilent Cary 5000 UV/vis/NIR spectrometer using PTFE reference disk as a standard. The IR spectra of the undoped and nitrogen-doped titania photocatalysts were measured on Thermoscientific spectrometer in the range of 4000–600 cm^{-1} in transmittance mode with a resolution of 16 cm^{-1} and 32 scans. X-ray photoelectron spectroscopy (XPS) measurements were performed using a Kratos Axis UltraDLD analysis system with monochromated Al Kα (hν = 1487 eV) X-ray source (15 kV, 10 mA). The survey scans were acquired with 160 eV pass energy and 1 eV step size. The high-resolution core level scans were acquired with 20 eV pass energy and 0.1 eV step size.

^{1}H and ^{13}C NMR spectra were recorded on a Bruker Avance NEO 400, Bruker Avance III 500, or Bruker Avance III 700 MHz spectrometer and were internally referenced to residual protio solvent signal (note: $CDCl_3$ referenced at δ 7.26 ppm for ^{1}H NMR and δ 77.16 ppm for ^{13}C NMR,

respectively). Data for ^{1}H NMR are reported as follows: chemical shift (δ ppm), multiplicity (s = singlet, d = doublet, t = triplet, q = quartet, m = multiplet, app=apparent), coupling constant (Hz), and integration. Data for ^{13}C NMR are reported in terms of chemical shift and no special nomenclature is used for equivalent carbons. High-resolution mass spectrometry data were recorded on an Agilent 7250 GC/Q-TOF using Agilent DB-5ms Ultra Inert column. The oven temperature starts at 50 °C and is held for 2 minutes then ramps at 10 °C/min until 300 °C and held at 300°C for 1 minute. The helium flow was kept at 1 mL/min. BET specific surface area analysis was carried out on Quantachrome Nova 4200e, the samples were degassed at 110 °C for four hours prior to analysis.

For EPR analysis, the Bl-SGHT-200-EtOH sample was prepared by loading 5 mg of SGHT-200 and 500 uL of ethanol in a glass vial stirred for 1 hour. Then, the suspension was transferred to EPR tube. The suspension was bubbled with N_2 under blue light (440 nm) and then EPR spectrum of titania suspension was acquired at 100 K with a Bruker EMX (X-band) spectrometer with the aid of a Bruker ER4111VT variable temperature unit. 10 scans were obtained using signal averaging with a microwave frequency of 9.44 GHz at a power of 2.02 mW while scanning 500G/84 s with a time constant of 164 ms. The receiver was set with a modulation amplitude of 5.14 G with a modulation frequency of 100-kHz, and a receiver gain of 5.02×10^{5}. The high-resolution TEM images were taken via Hitachi HT-7800 transmission electron microscope using formvar/carbon supported copper grids (grid size 400 mesh) as support. The average particle size was estimated by counting 150 particles.

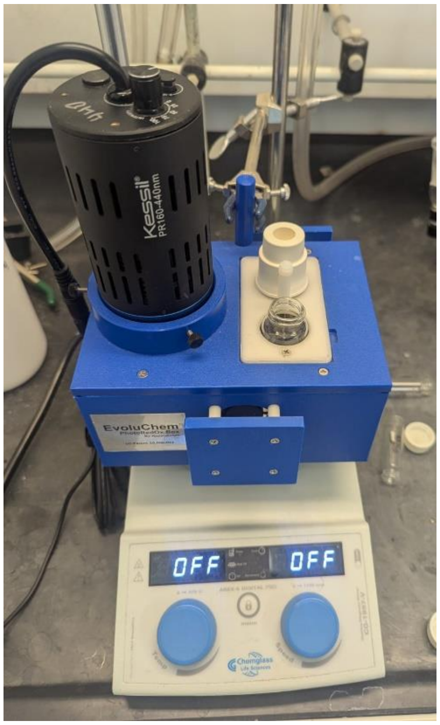

Figure S1 Photocatalytic set up, Hepato Chem, EvoluChemTM PhotoRedOx Box.

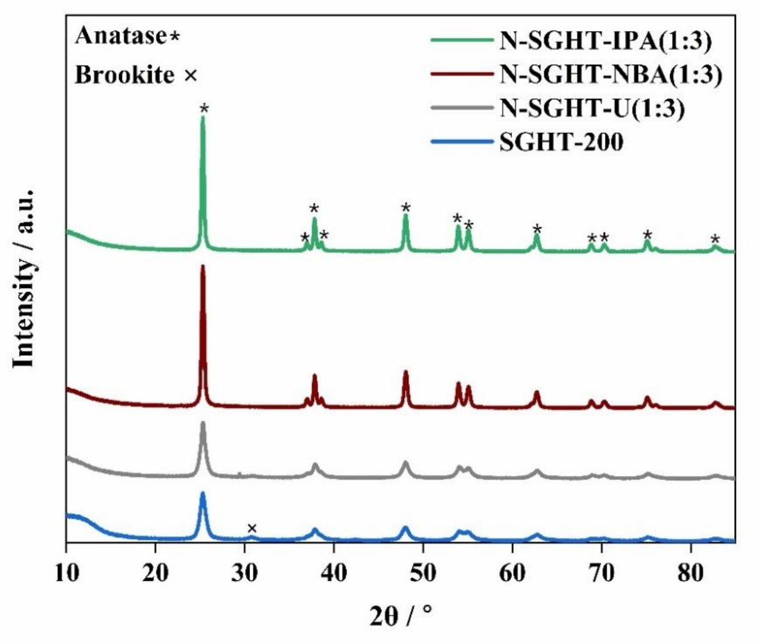

Figure S2 X-ray diffraction (XRD) patterns of undoped titania and nitrogen-doped titania photocatalysts.

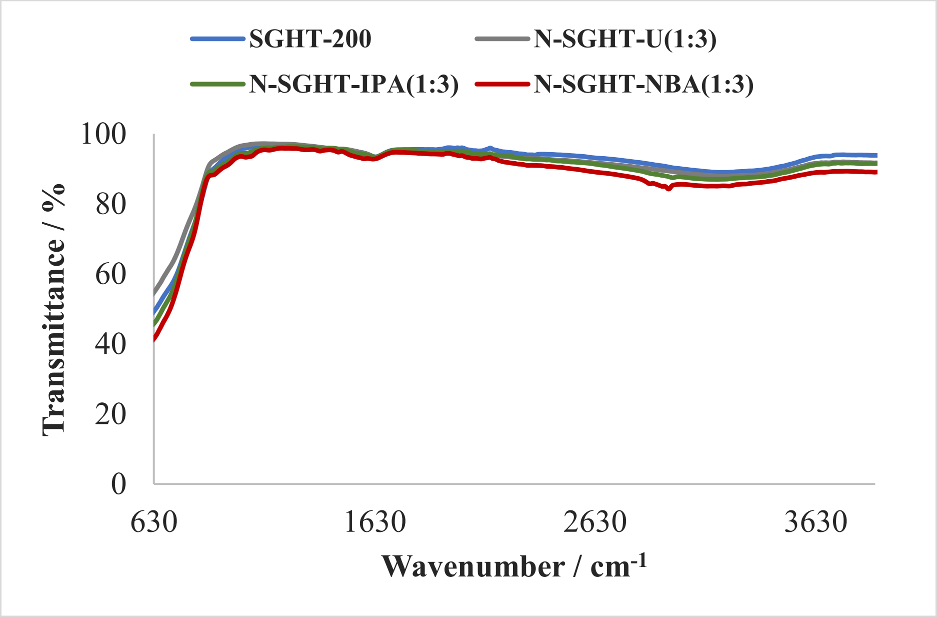

Figure S3 IR spectrum of the titania and nitrogen-doped titania samples.

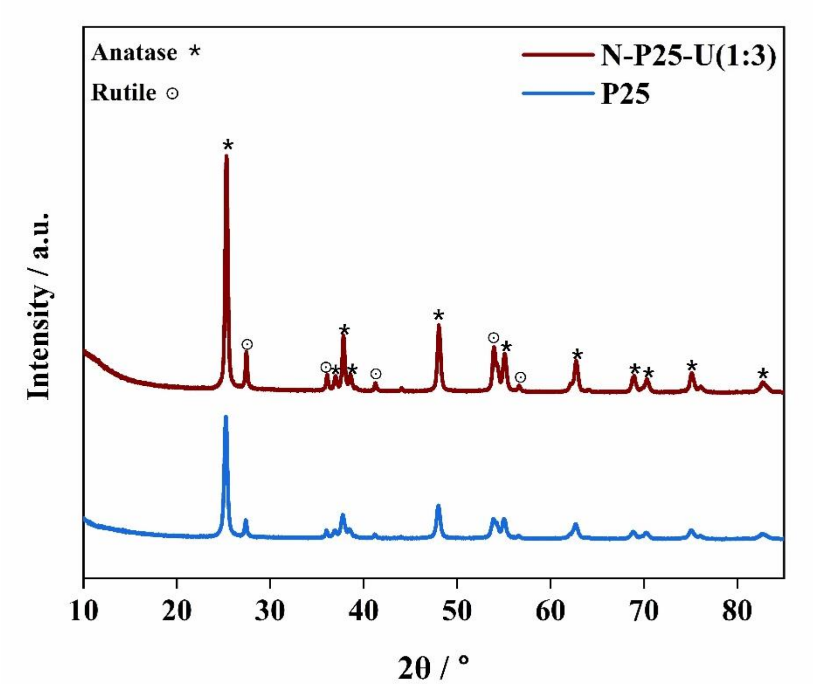

Figure S4 X-ray diffraction (XRD) patterns of undoped commercial titania (P25) and nitrogen-doped commercial titania photocatalysts.

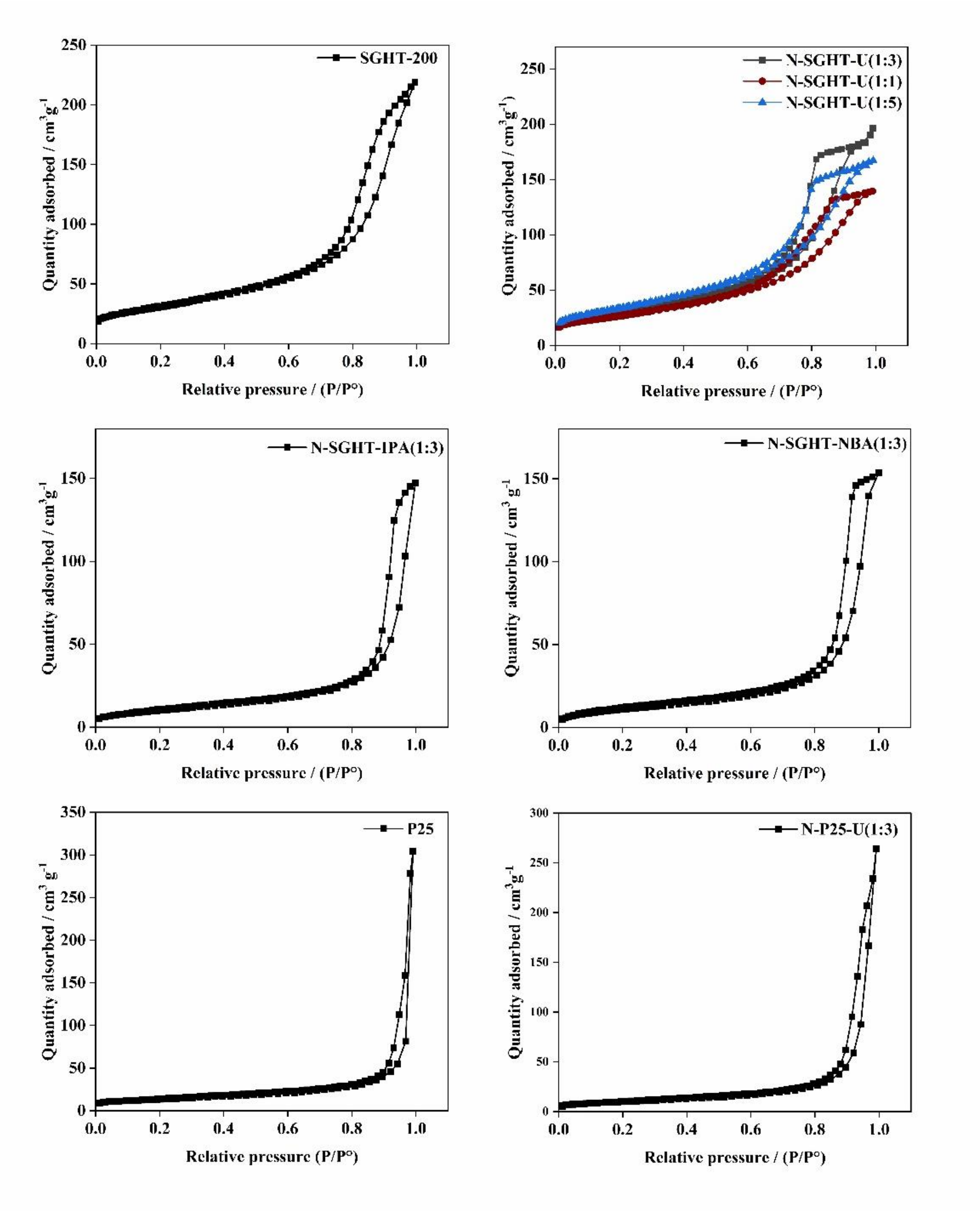

Figure S5 Nitrogen adsorption-desorption isotherms of undoped titania and nitrogen-doped titania photocatalysts.

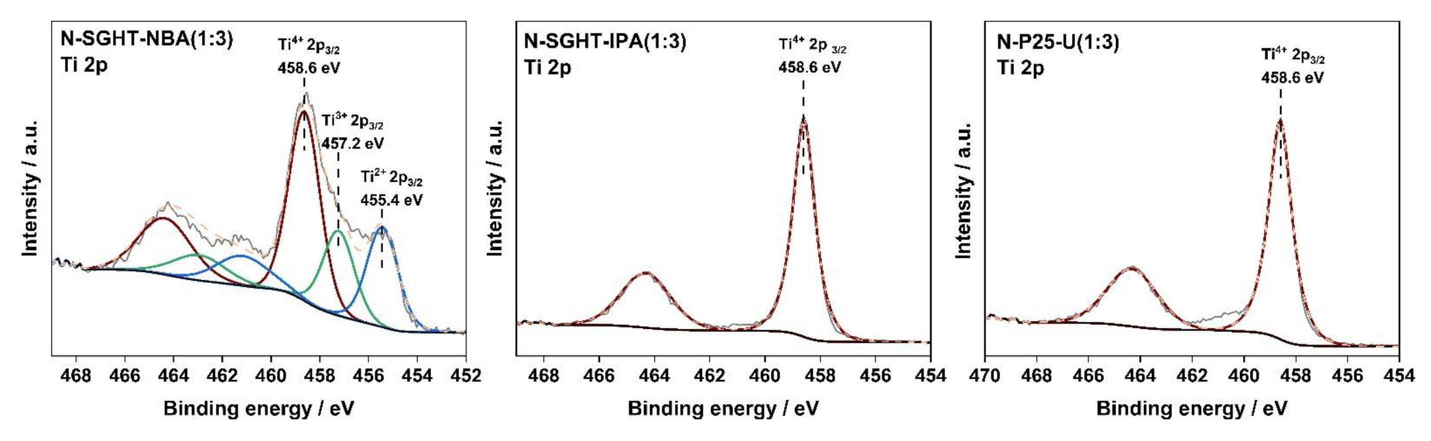

Figure S6 XPS Ti 2p spectra of nitrogen-doped titania photocatalysts.

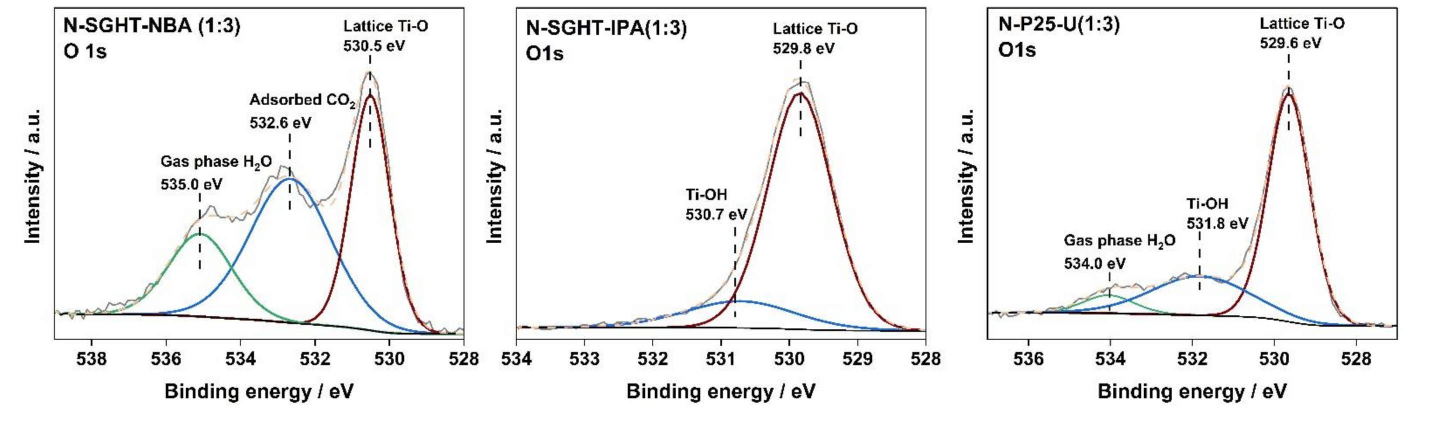

Figure S7 XPS O 1s spectra of nitrogen-doped titania photocatalysts.

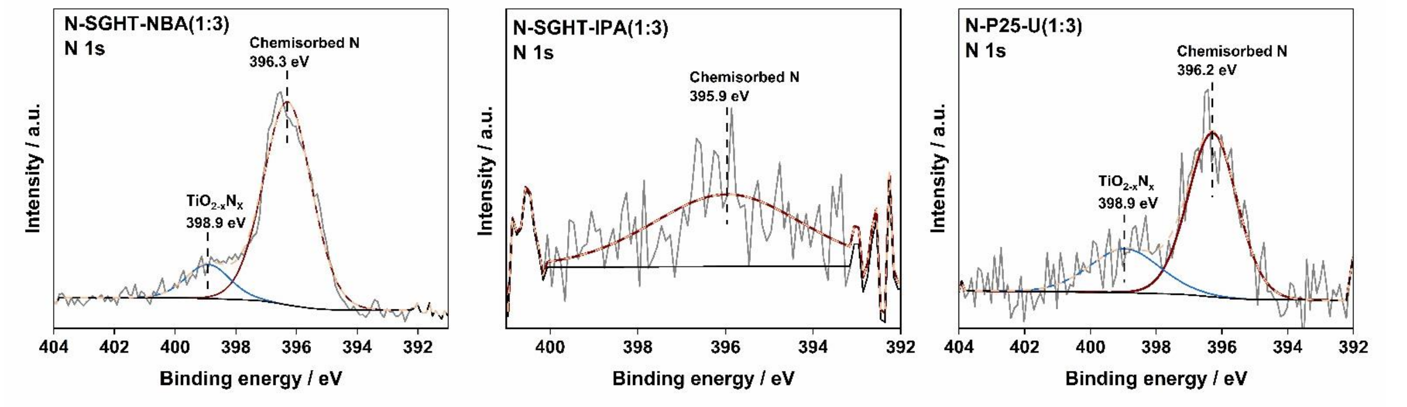

Figure S8 XPS N 1s spectra of nitrogen-doped titania photocatalysts.

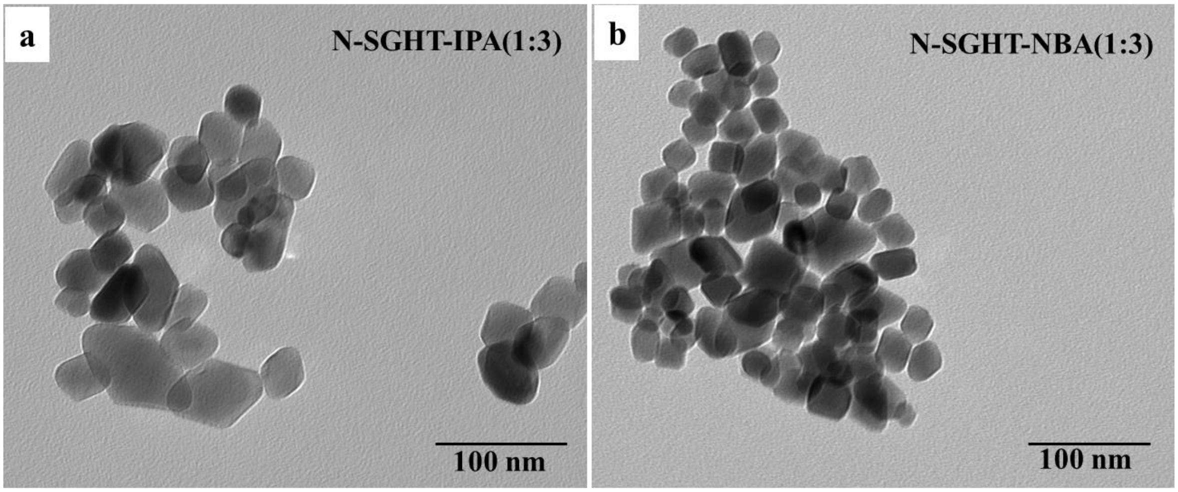

Figure S9 High resolution TEM images of a) nitrogen-doped titania prepared using *iso*-propylamine as nitrogen dopant b) nitrogen-doped titania prepared using *n*-butylamine as nitrogen dopant.

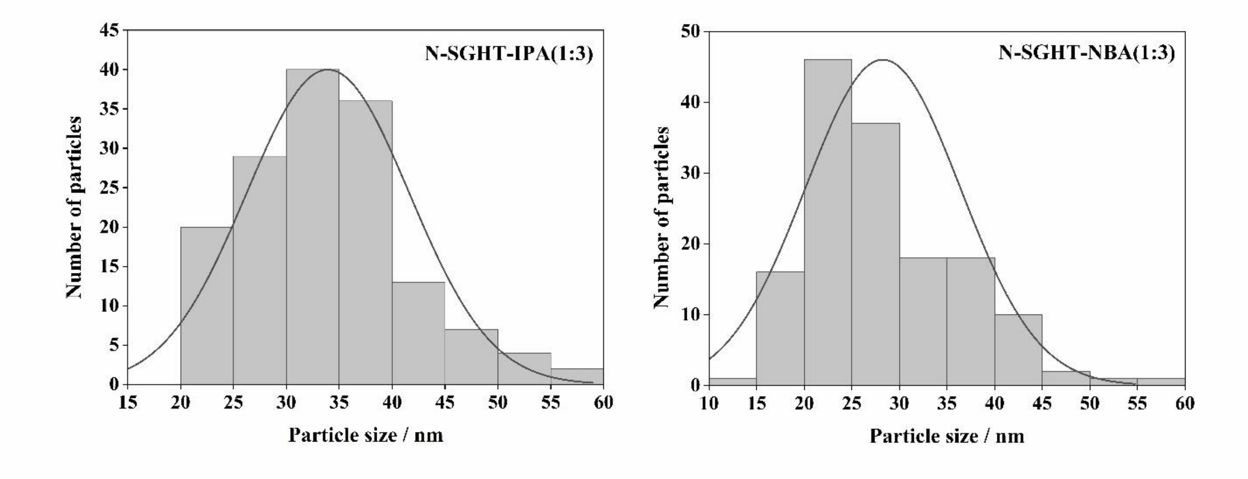

Figure S10 Particle size distribution of nitrogen-doped titania photocatalysts.

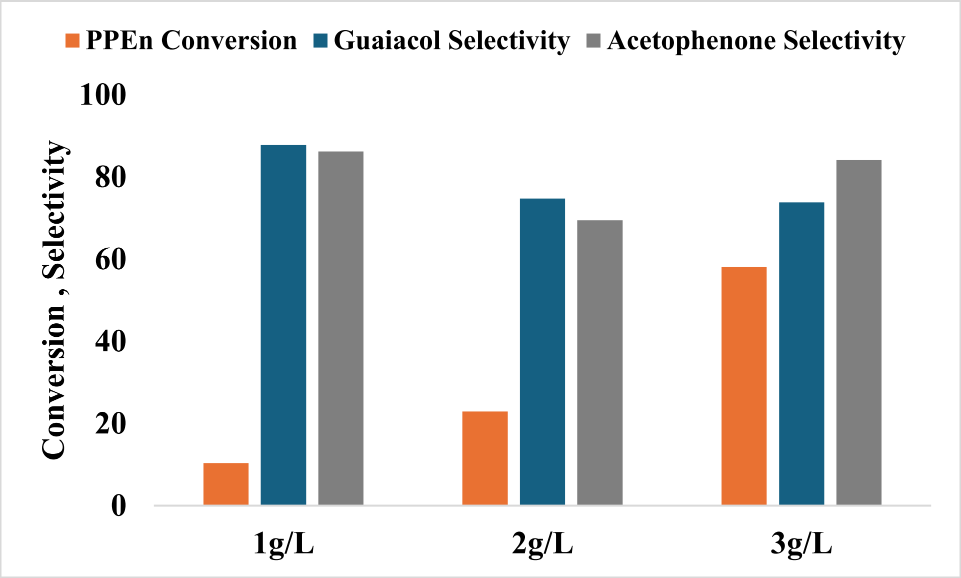

Figure S11 Effect of catalyst loading on the photocatalytic performance of SGHT-200 in the reductive cleavage of 2-(2-methoxyphenoxy)-1-phenylethanone (PPEn) under blue light (440 nm).

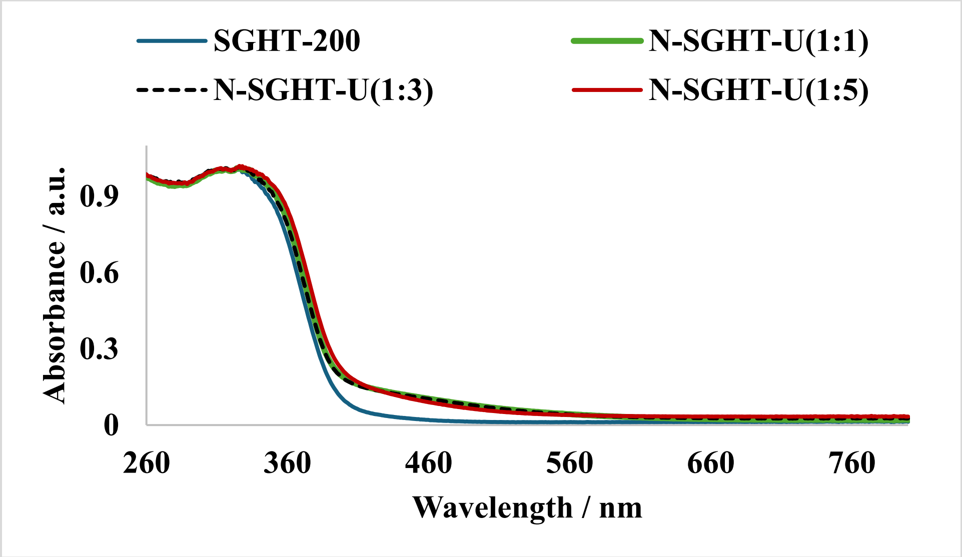

Figure S12 DRS UV-visible absorption spectra of nitrogen-doped titania prepared using urea as a nitrogen dopant source.

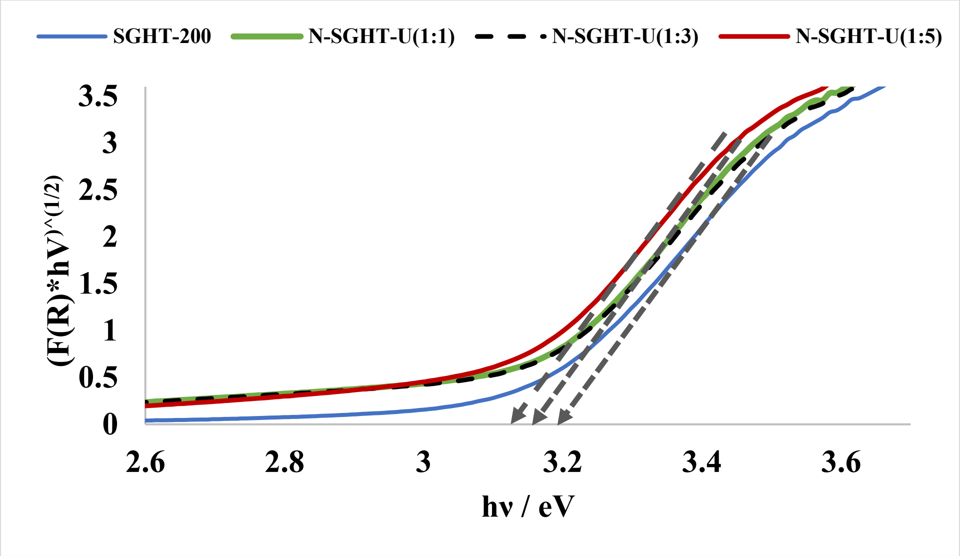

Figure S13 Tauc plots of nitrogen-doped titania prepared using urea as a nitrogen dopant source.

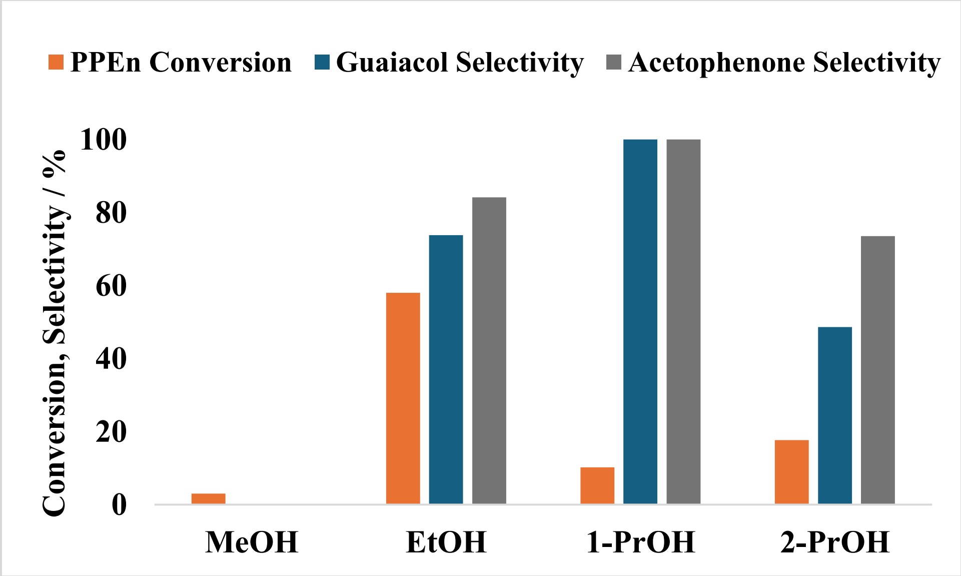

Figure S14 Effect of solvent on the photocatalytic performance of SGHT-200 in the reductive cleavage of 2-(2-methoxyphenoxy)-1-phenylethanone (PPEn) under blue light (440 nm).

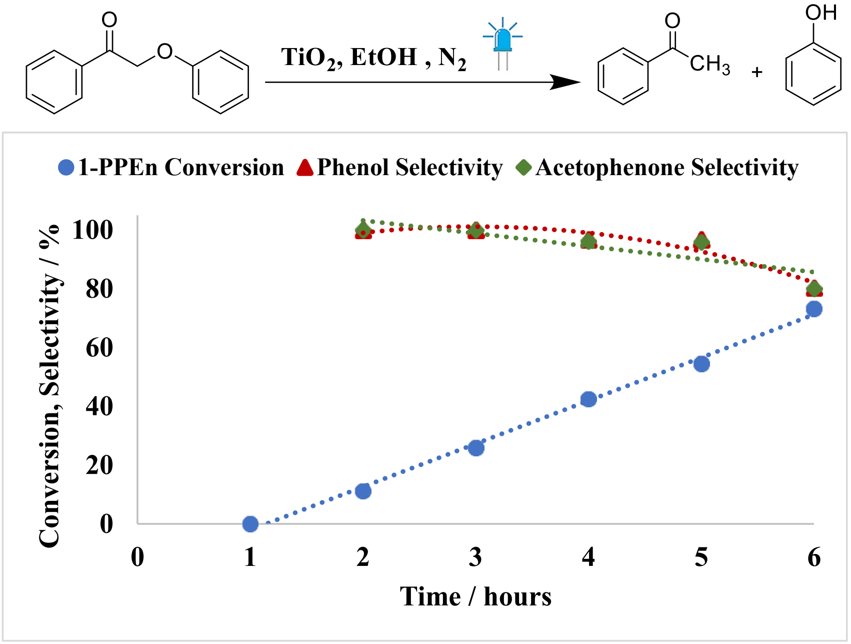

Figure S15 Effect of reaction time on the photocatalytic performance of SGHT-200 (3g/L) in the reductive cleavage of 2-phenoxy-1-phenylethanone (1-PPEn) under blue light (440 nm).

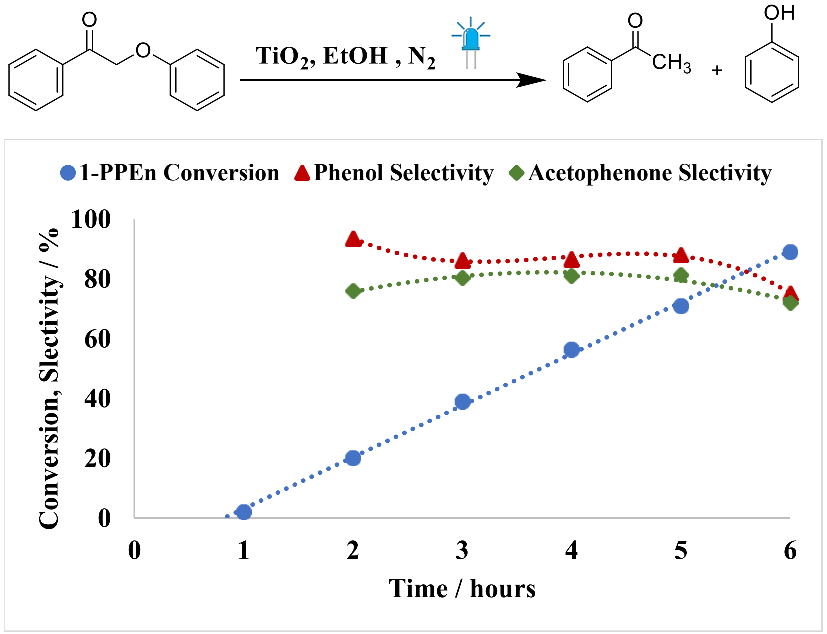

Figure S16 Effect of reaction time on the photocatalytic performance of N-SGHT-U(1:3) (3g/L) in the reductive cleavage of 2-phenoxy-1-phenylethanone (1-PPEn) under blue light (440 nm).

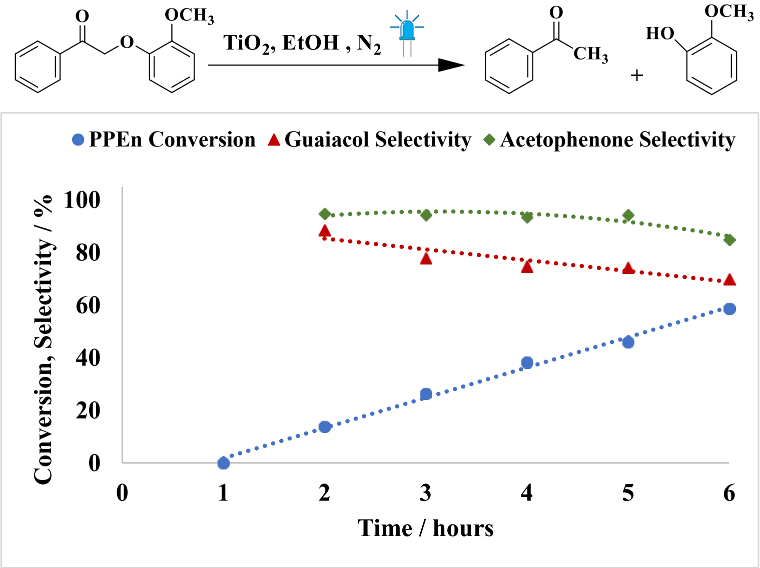

Figure S17 Effect of reaction time on the photocatalytic performance of SGHT-200 (3g/L) in the reductive cleavage of 2-(2-methoxyphenoxy)-1-phenylethanone (PPEn) under blue light (440nm).

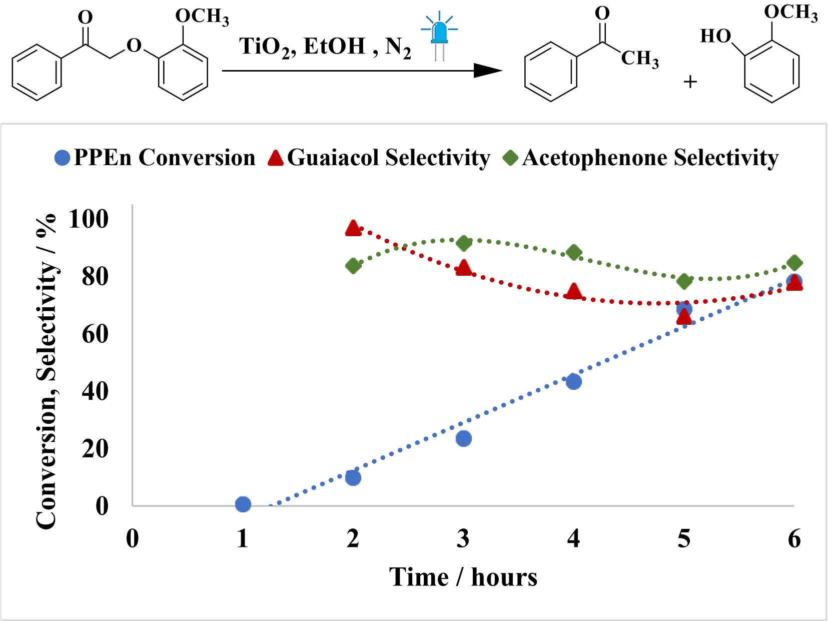

Figure S18 Effect of reaction time on the photocatalytic performance of N-SGHT-U(1:3) (3g/L) in the reductive cleavage of 2-(2-methoxyphenoxy)-1-phenylethanone (PPEn) under blue light (440nm).

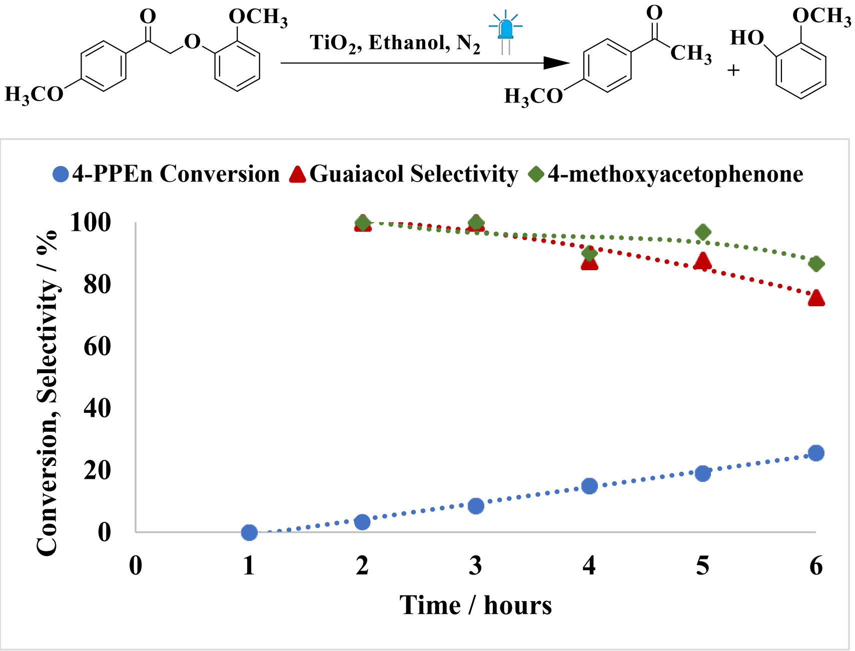

Figure S19 Effect of reaction time on the photocatalytic performance of SGHT-200 (3g/L) in the reductive cleavage of 2-(2-methoxyphenoxy)-1-(4-methoxyphenyl)ethanone (4-PPEn) under blue light (440nm).

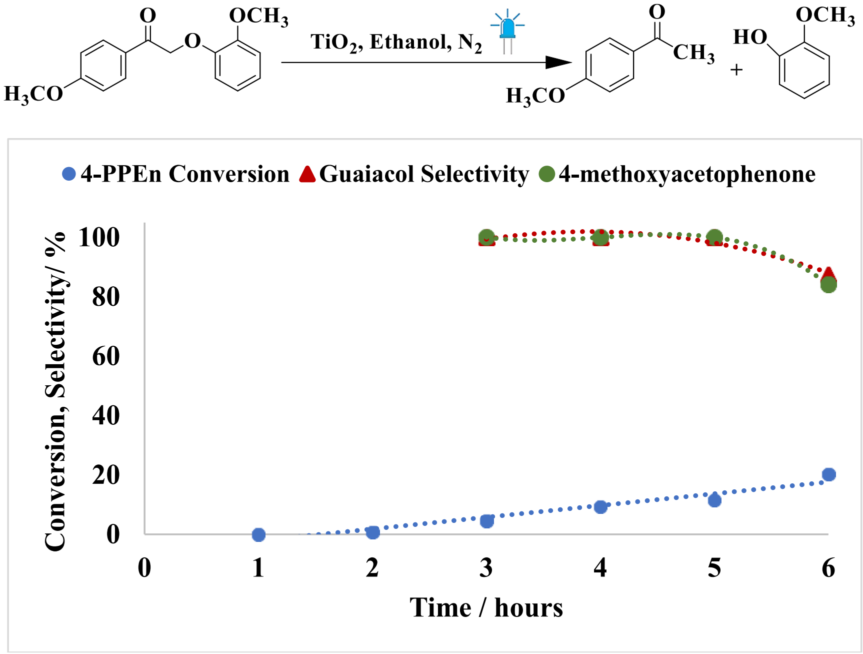

Figure S20 Effect of reaction time on the photocatalytic performance of N-SGHT-U(1:3) (3g/L) in the reductive cleavage of 2-(2-methoxyphenoxy)-1-(4-methoxyphenyl)ethanone (4-PPEn) under blue light (440nm).

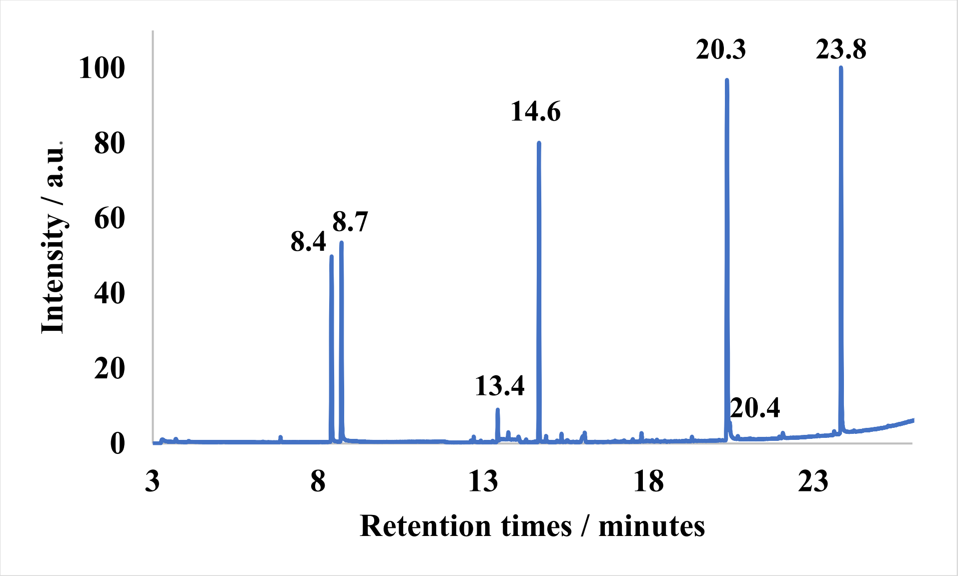

Figure S21 GC chromatograms of photocatalytic reductive cleavage of β-O-4 ketone (PPEn) by SGHT-200 in ethanol under blue light (440 nm).

The peaks detected at 8.4 minutes, 8.7 minutes, 13.4 minutes, 14.6 minutes, 20.3 minutes, 20.4 minutes and 23.8 minutes are assigned to acetophenone, guaiacol, diphenyl ether, butylated hydroxytoluene, 2-(2-Methoxyphenoxy)-1-phenylethanone, 2-(2-Methoxyphenoxy)-1-phenylethanol, 2,2'-methylenebis[4-methyl-6-tert-butylphenol], respectively. The remaining very small peaks at 3.2 minutes, 16.0 minutes, 17.7 minutes are related to column bleed.

Note: diphenyl ether, butylated hydroxytoluene, 2,2'-methylenebis[4-methyl-6-tert-butylphenol] are observed due to contamination from septum.

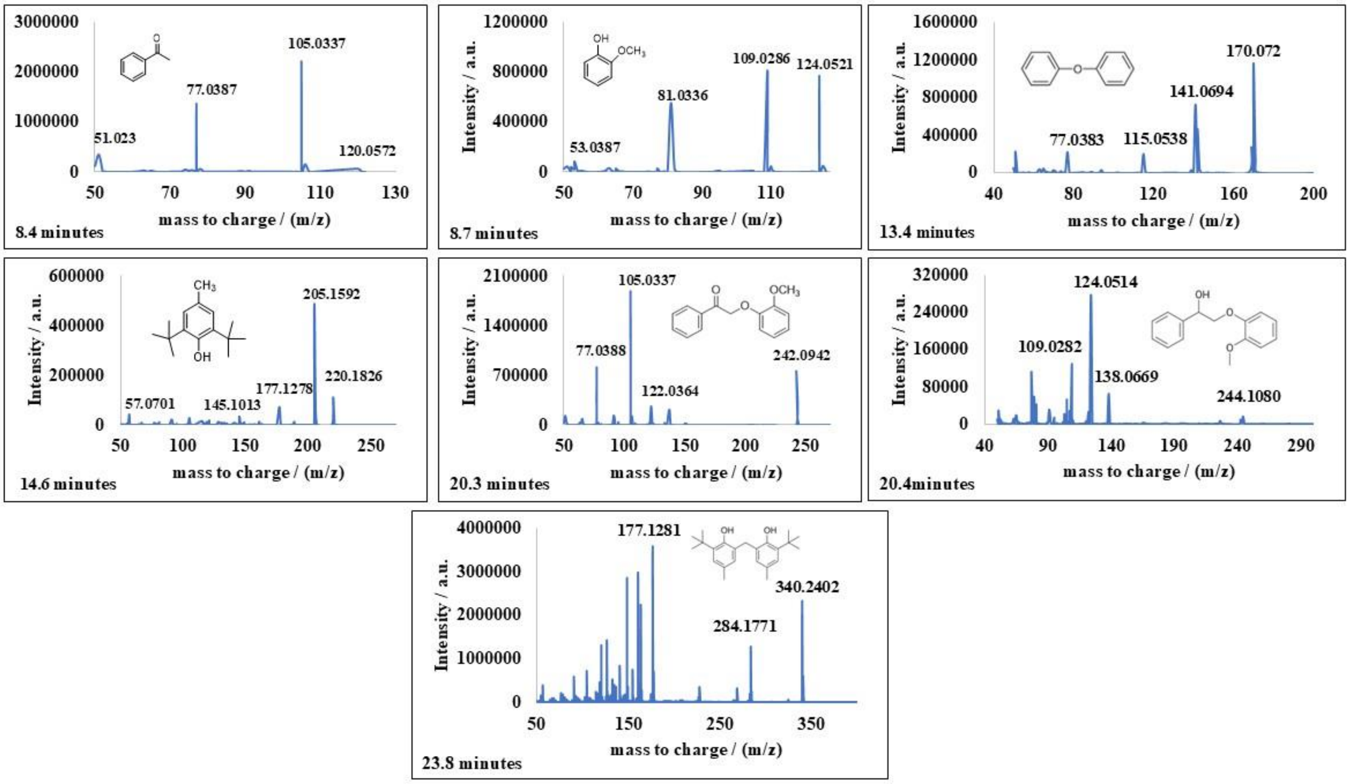

Figure S22 Mass spectrum of compounds detected in the photocatalytic reductive cleavage of β-O-4 ketone (PPEn) by SGHT-200 in ethanol under blue light (440 nm).

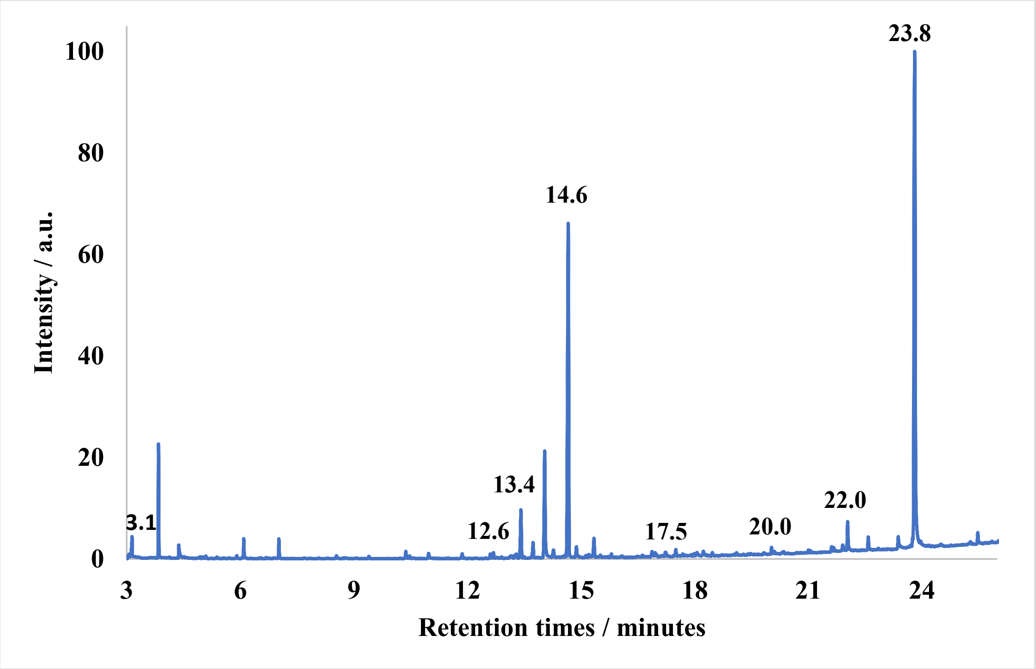

Figure S23 GC chromatogram for the photocatalytic reductive cleavage of ethanosolv lignin by SGHT-200 under blue light (440 nm).

The peaks detected at 3.1 minutes, 12.6 minutes, 13.4 minutes, 14.6 minutes, 17.5 minutes, 20.0 minutes, 22.0 minutes and 23.8 minutes are assigned to acetic acid, phenol, 2-(1,1-dimethylethyl)-4-methyl-, diphenyl ether, butylated hydroxytoluene, benzene, 1-(1,1-dimethylethyl)-4-phenoxy-, ethyl palmitate, acetic acid n-octadecyl ester, 2,2'-methylenebis[4-methyl-6-tert-butylphenol], respectively.

Note: diphenyl ether, butylated hydroxytoluene, 2,2'-methylenebis[4-methyl-6-tert-butylphenol] are observed due to contamination from septum.

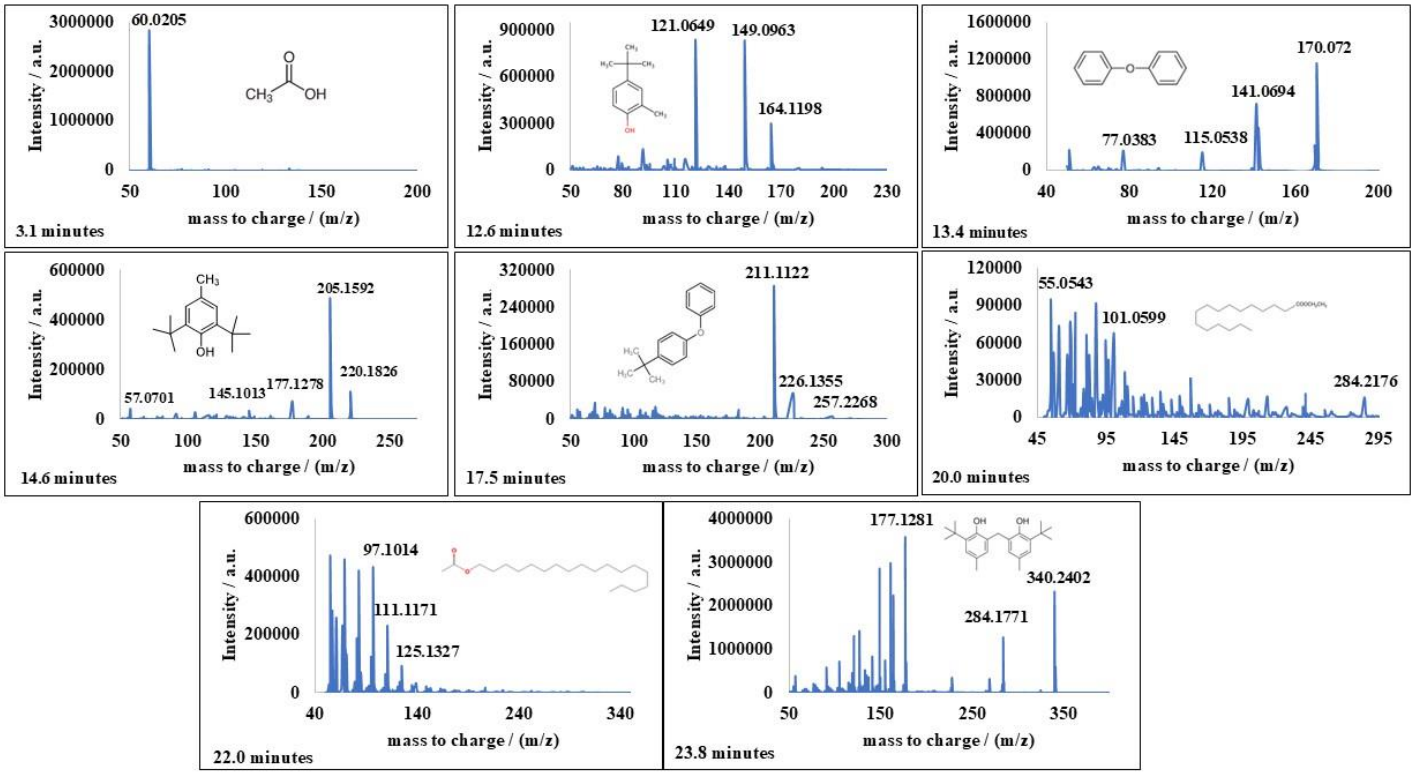

Figure S24 Mass spectrum of compounds detected in the photocatalytic cleavage of ethanosolv lignin in ethanol under blue light (440 nm).

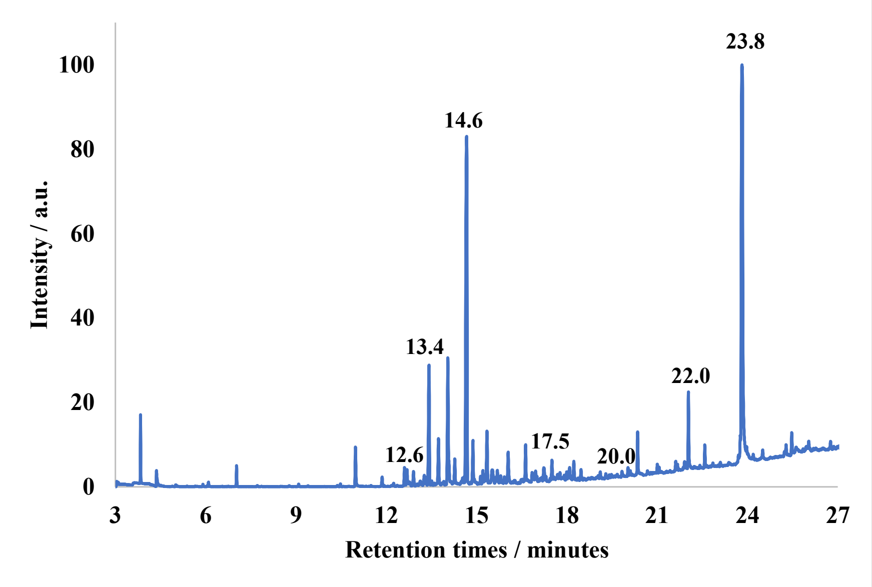

Figure S25 GC chromatogram for photolysis of ethanosolv lignin under blue light (440 nm).

The peaks detected at 12.6 minutes, 13.4 minutes, 14.6 minutes, 17.5 minutes, 20.0 minutes, 22.0 minutes and 23.8 minutes are assigned to phenol, 2-(1,1-dimethylethyl)-4-methyl-, diphenyl ether, butylated hydroxytoluene, benzene, 1-(1,1-dimethylethyl)-4-phenoxy-, ethyl palmitate, acetic acid n-octadecyl ester, 2,2'-methylenebis[4-methyl-6-tert-butylphenol], respectively.

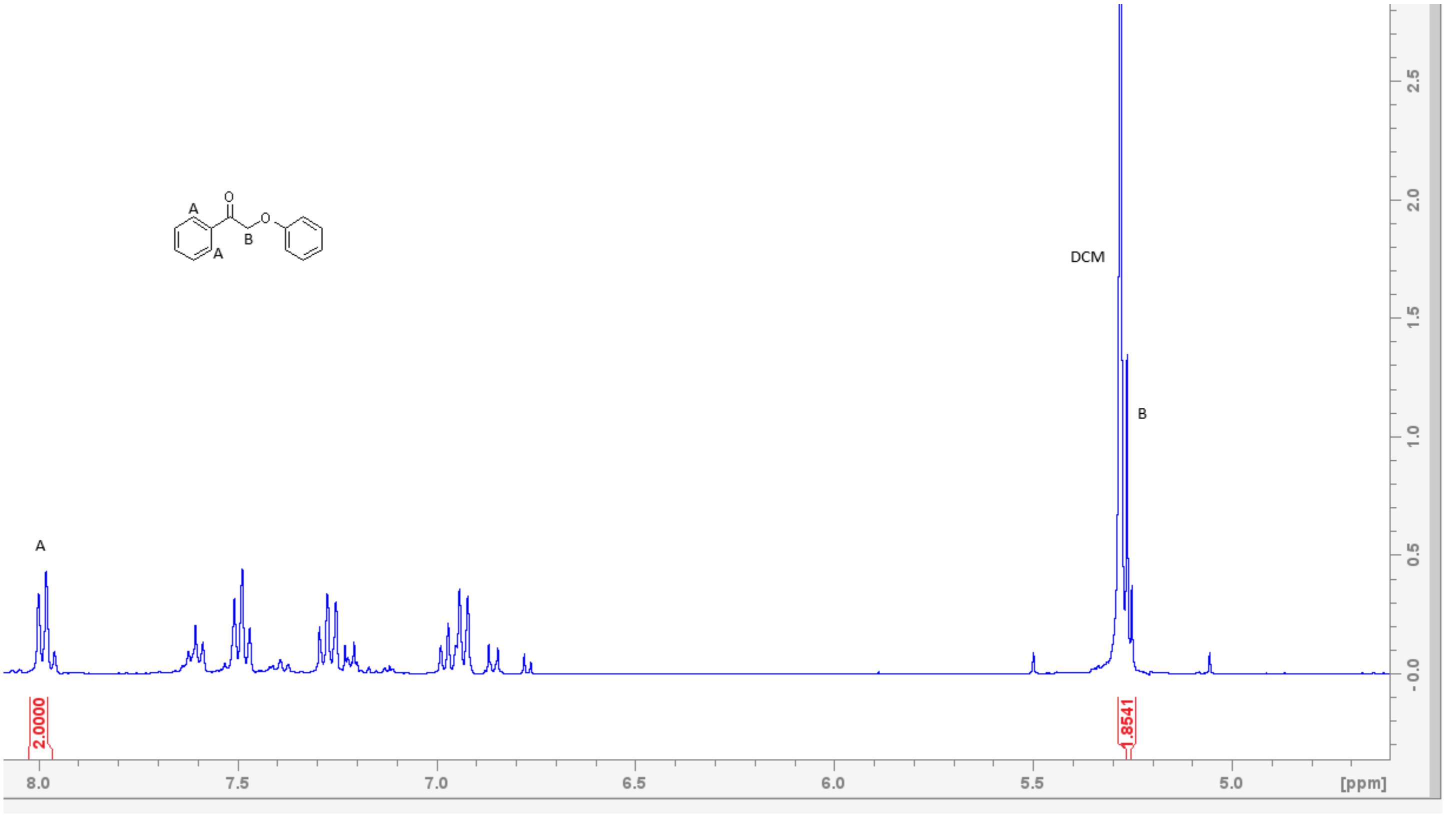

Figure S26 ^{1}H NMR spectra for 2-phenoxy-1-phenylethanone.

The aromatic peak at 7.99 ppm labeled "A" has shifted downfield compared to the starting material (7.45ppm), due to the incorporation of the carbonyl group.[2] We also see the appearance of the methylene singlet (labeled "B") at 5.26 ppm, mildly covered by dichloromethane, which is indicative of removing the neighboring proton in the starting material. No starting material peaks are found to be present in the crude mixture, specifically the benzylic hydrogen alpha to the alcohol which typically appears as a doublet of triplets at 5.00 ppm.[2] This indicated to us that the method was exceptional at this oxidation, reaching quantitative conversion of the starting material, and could be applied to the oxidation of ethanosolv lignin.

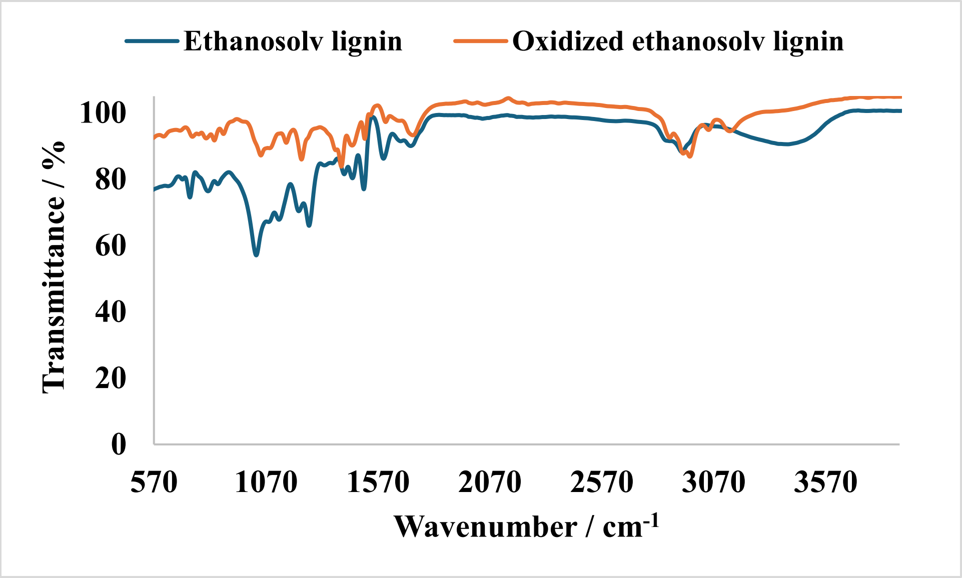

Figure S27 IR spectrum of as obtained ethanosolv lignin and oxidized ethanosolv lignin.

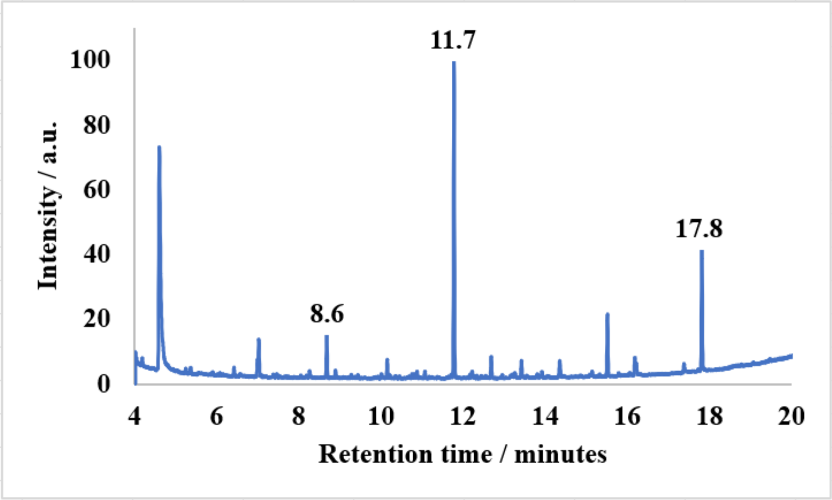

Figure S28 GC chromatogram for the photocatalytic reductive cleavage of oxidized ethanosolv lignin by SGHT-200 under blue light (440 nm).

The peaks detected at 8.6 minutes, 11.7 minutes, 17.8 minutes, butylated hydroxytoluene, 5-isopropyl-2-methylphenyl heptanoate, 2,2'-methylenebis[4-methyl-6-tert-butylphenol], respectively.

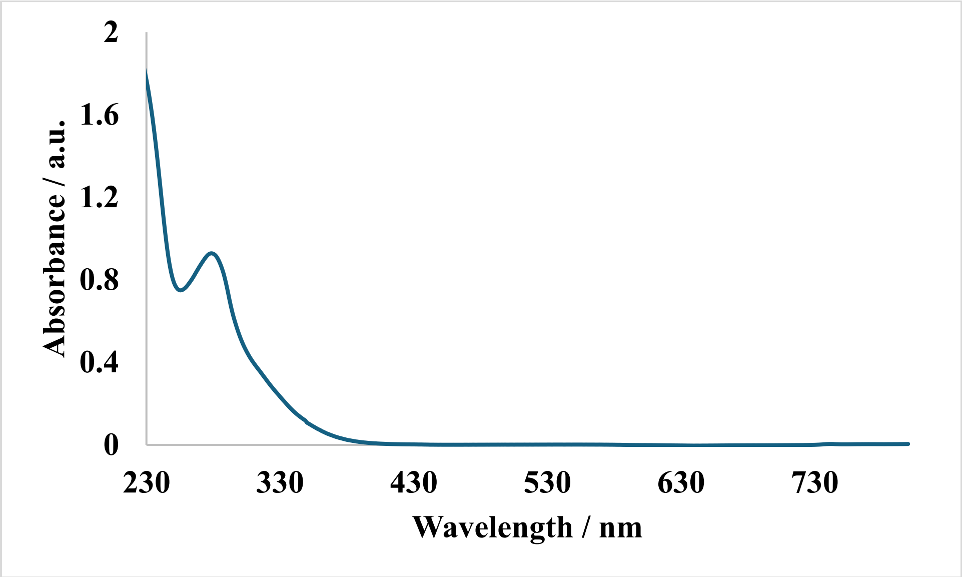

Figure S29 UV-visible absorption spectrum of ethanosolv lignin in ethanol.

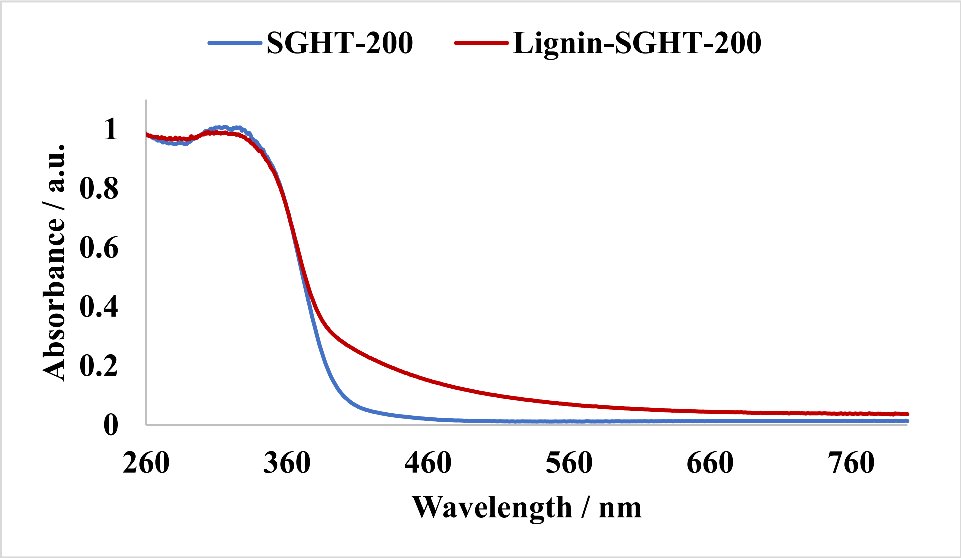

Figure S30 DRS UV-visible absorption spectrum of SGHT-200 exposed to unmodified ethanosolv lignin solution in ethanol.

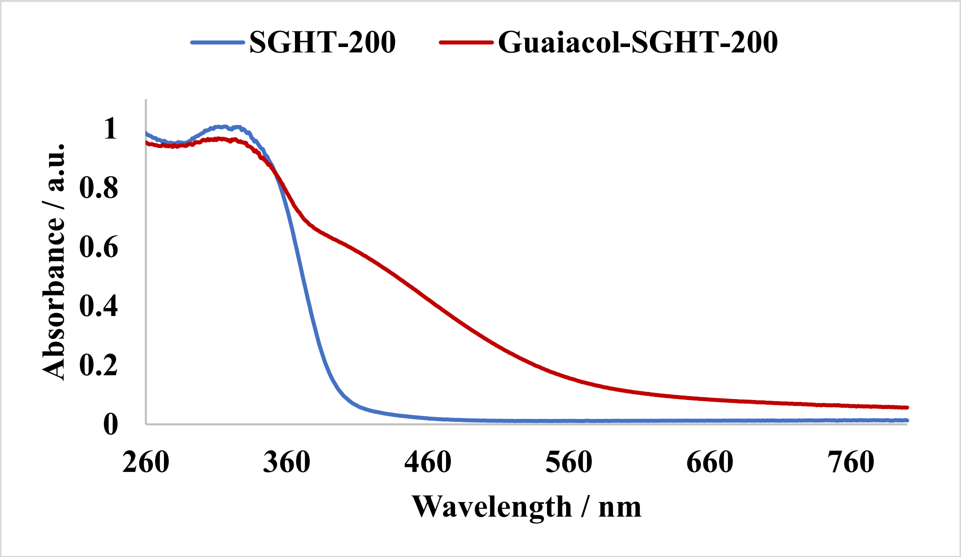

Figure S31 DRS UV-visible absorption spectrum of guaiacol adsorbed SGHT-200.

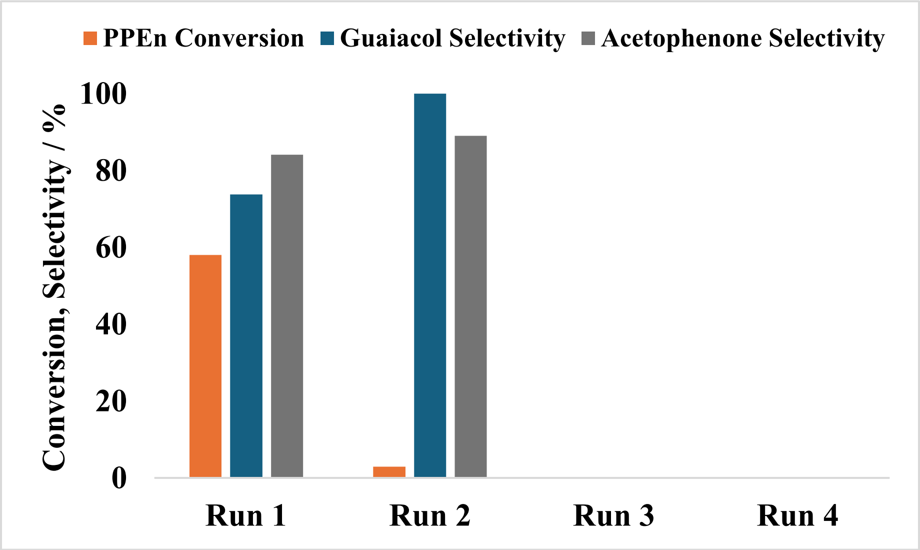

Figure S32 Recyclability test for SGHT-200 for the reductive cleavage of 2-(2-methoxyphenoxy)-1-phenylethanone (PPEn) under blue light (440 nm).

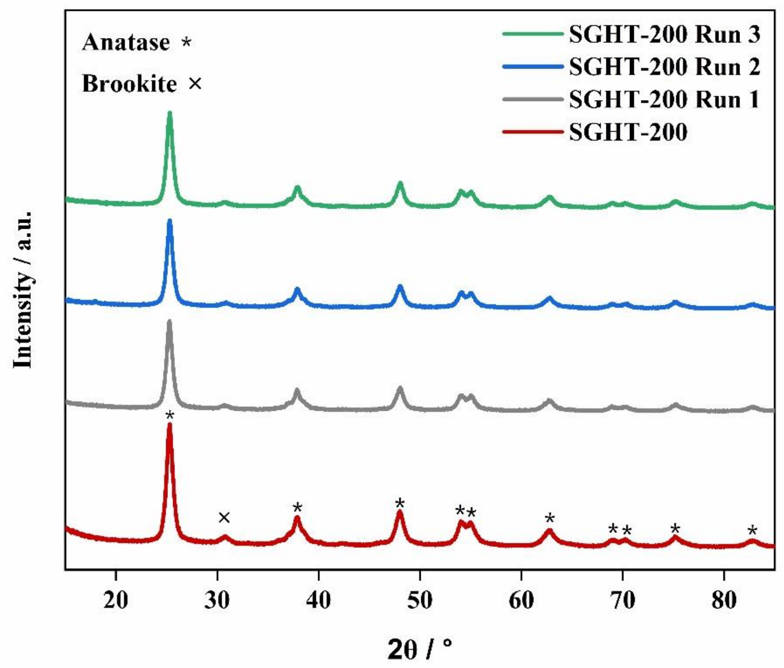

Figure S33 X-ray diffraction (XRD) patterns of SGHT-200 reused in reductive cleavage of 2-(2-methoxyphenoxy)-1-phenylethanone (PPEn) under blue light (440 nm).

Table S1 Crystallographic features and textural properties (Brunauer-Emmett-Teller specific surface area [SSA], Barrett-Joyner-Halenda [BJH] pore volume and pore radius) of SGHT-200 reused in reductive cleavage of 2-(2-methoxyphenoxy)-1-phenylethanone (PPEn) under blue light (440 nm).

Entry	Catalyst	Ratio of crystalline phases (%)	Crystal size (nm)		SSA (m^2g^{-1})	BJH pore radius (Å)	BJH pore volume (cm^3g^{-1})
		Anatase:Brookite	Anatase	Brookite			
1	SGHT-200	79:21	11	8	109	55	0.335
2	SGHT-200 Run 1	85:15	12	8	92	55	0.322
3	SGHT-200 Run 2	80:20	11	6	89	50	0.332
4	SGHT-200 Run 3	86:14	12	8	75	55	0.315
5	SGHT-200 Run 4	86:14	12	8	86	50	0.315

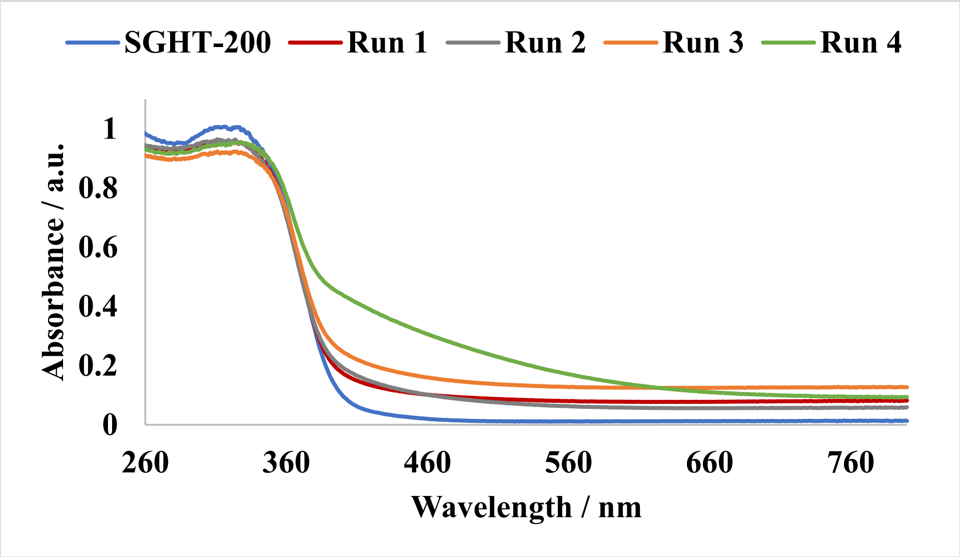

Figure S34 DRS UV-visible absorption spectrum of SGHT-200 reused in reductive cleavage of 2-(2-methoxyphenoxy)-1-phenylethanone (PPEn) under blue light (440 nm).

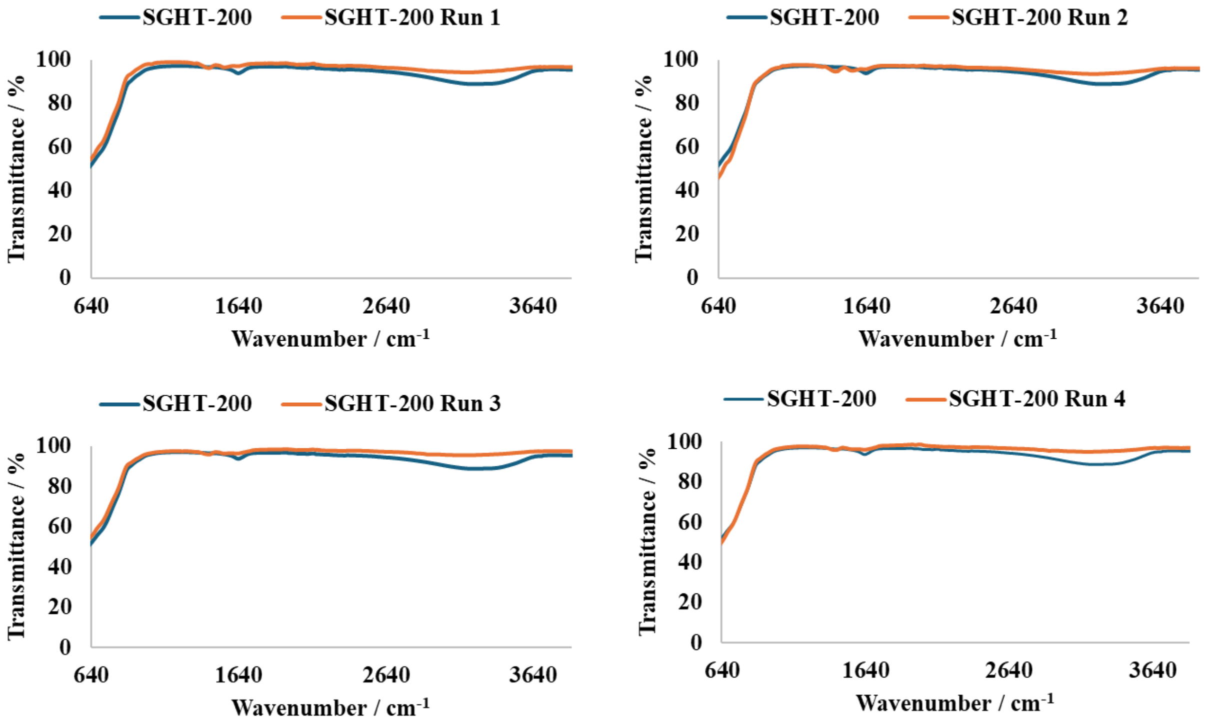

Figure S35 IR analysis of SGHT-200 reused in the reductive cleavage of 2-(2-methoxyphenoxy)-1-phenylethanone (PPEn) under blue light (440 nm) and washed with acetonitrile.

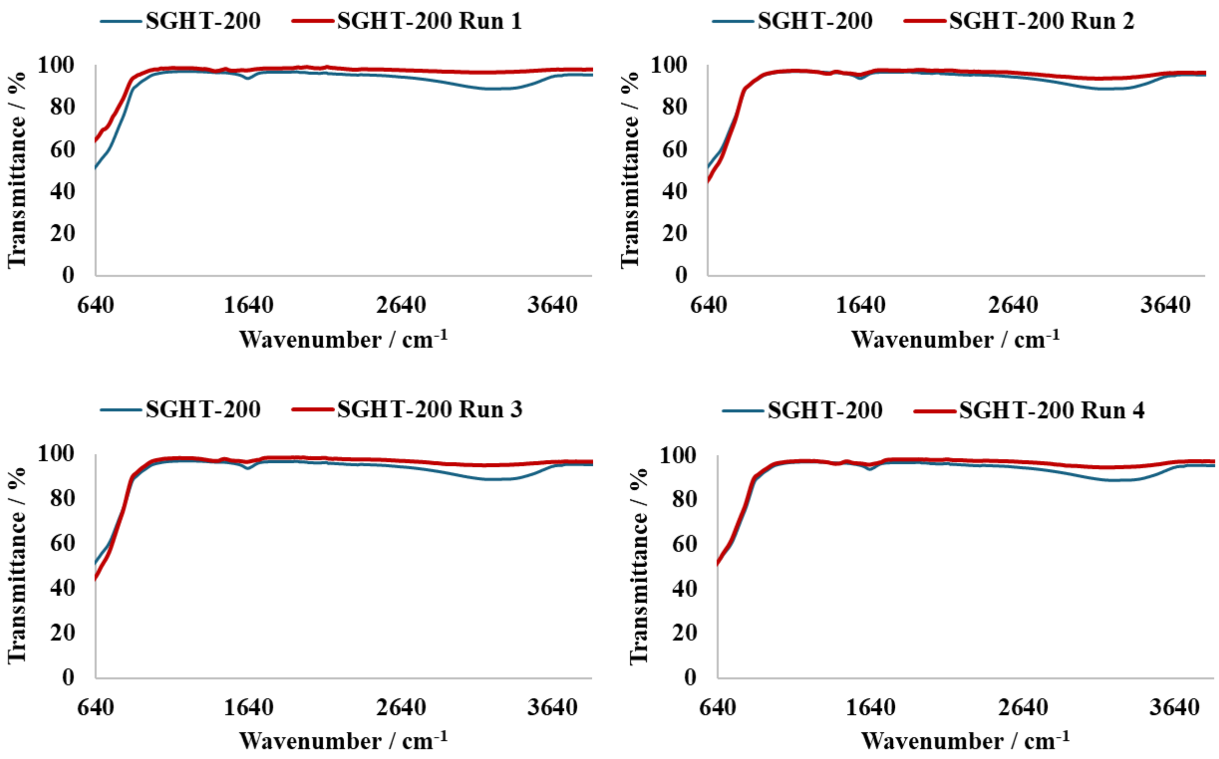

Figure S36 IR analysis of SGHT-200 reused in the reductive cleavage of 2-(2-methoxyphenoxy)-1-phenylethanone (PPEn) under blue light (440 nm) and washed with water.

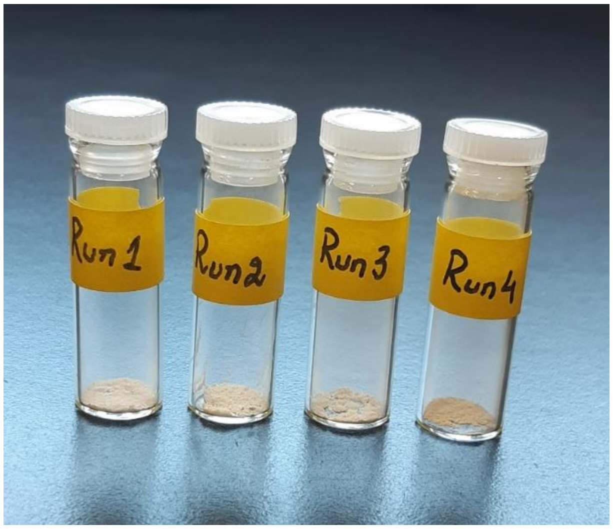

Figure S37 SGHT-200 after multiple runs in reductive cleavage of 2-(2-methoxyphenoxy)-1-phenylethanone (PPEn) under blue light (440 nm).

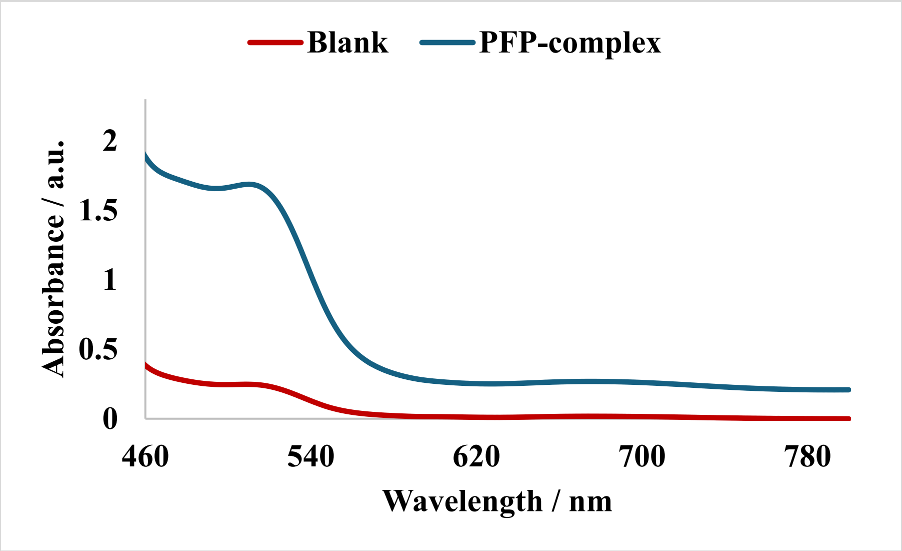

Figure S38 UV-visible absorption spectrum of potassium–ferrioxalate phenanthroline (PFP) complex after blue light (440 nm) irradiation.

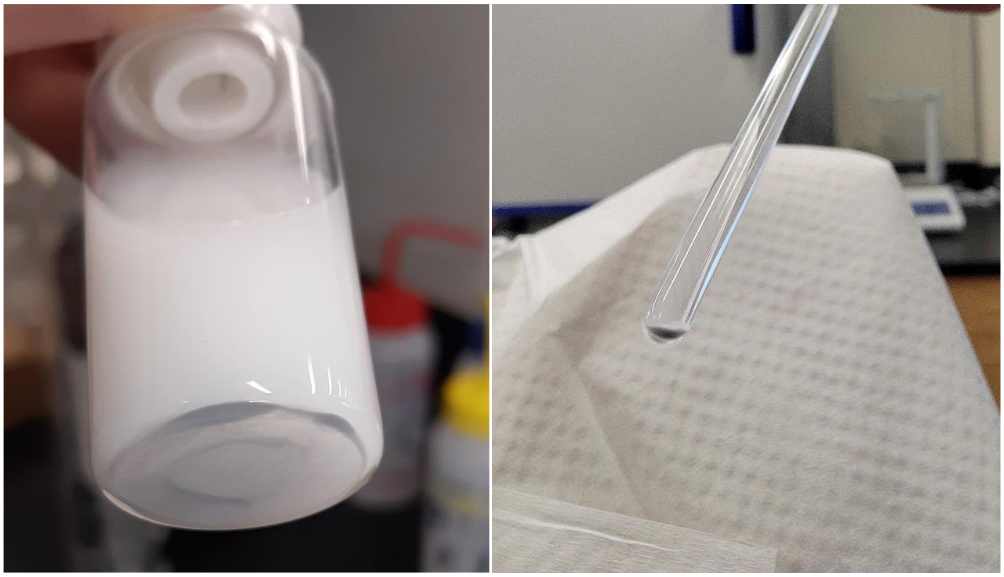

Figure S39 Titania suspension in ethanol irradiated under blue light (440 nm) in photoreactor (left panel) and in EPR tube (right panel)

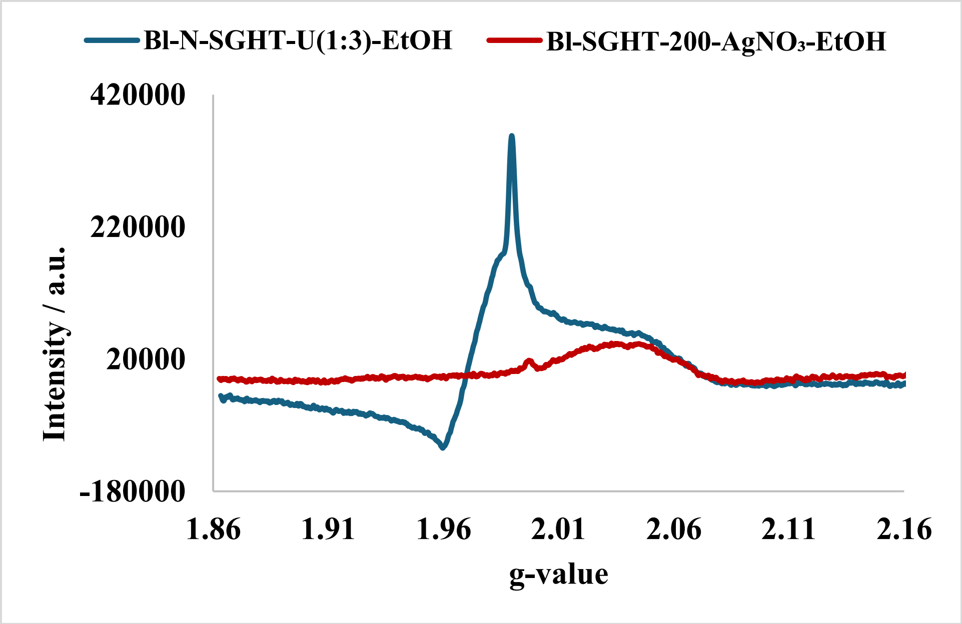

Figure S40 EPR spectrum of N-SGHT-U(1:3) suspension in ethanol and SGHT-200 in ethanol in the presence of silver(I) nitrate after blue light irradiation (440 nm).

Note: No Ti^{3+} signal observed in the presence of electron acceptor, silver(I)nitrate

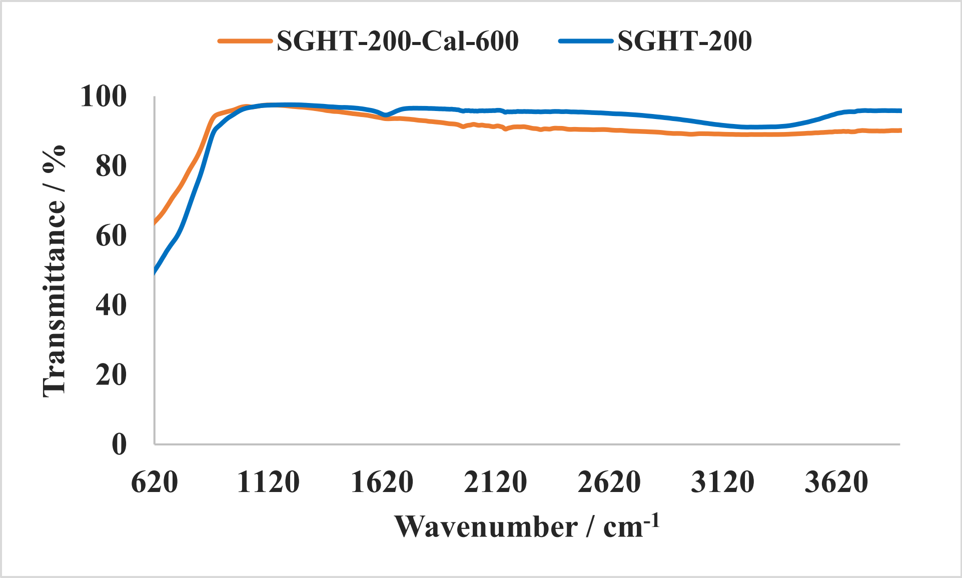

Figure S41 IR spectra of SGHT-200 and calcined titania (SGHT-200-Cal-600).

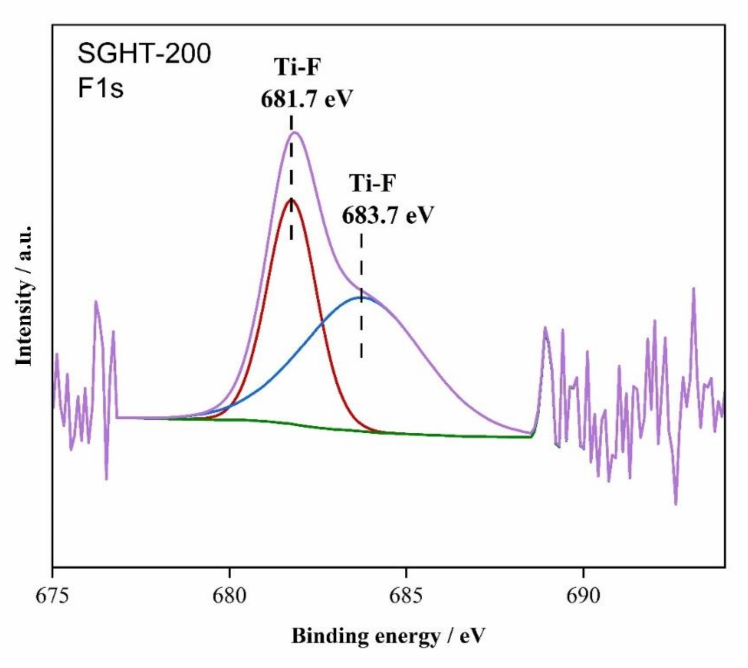

Figure S42 F1s XPS spectra of fluorinated SGHT-200 (F-SGHT-200).

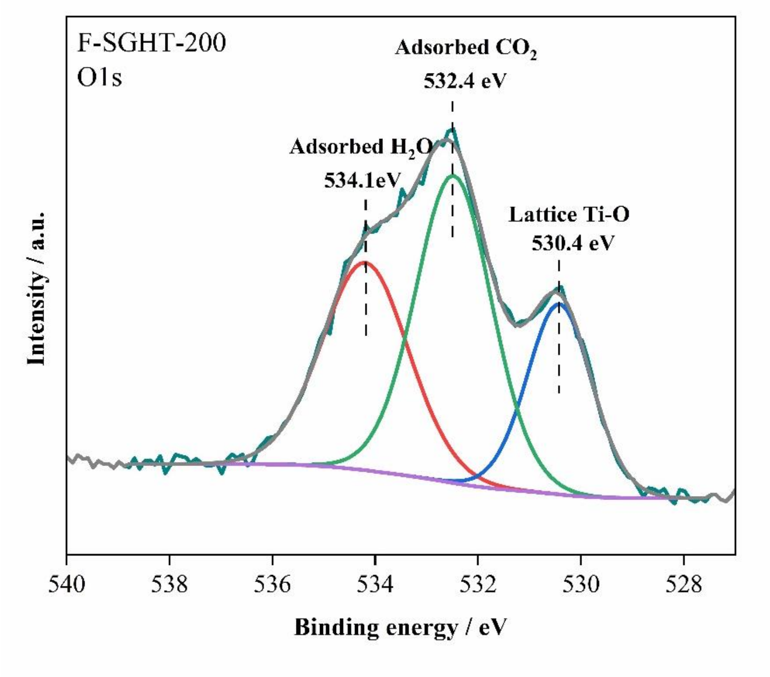

Figure S43 O1s XPS spectra of fluorinated SGHT-200 (F-SGHT-200).

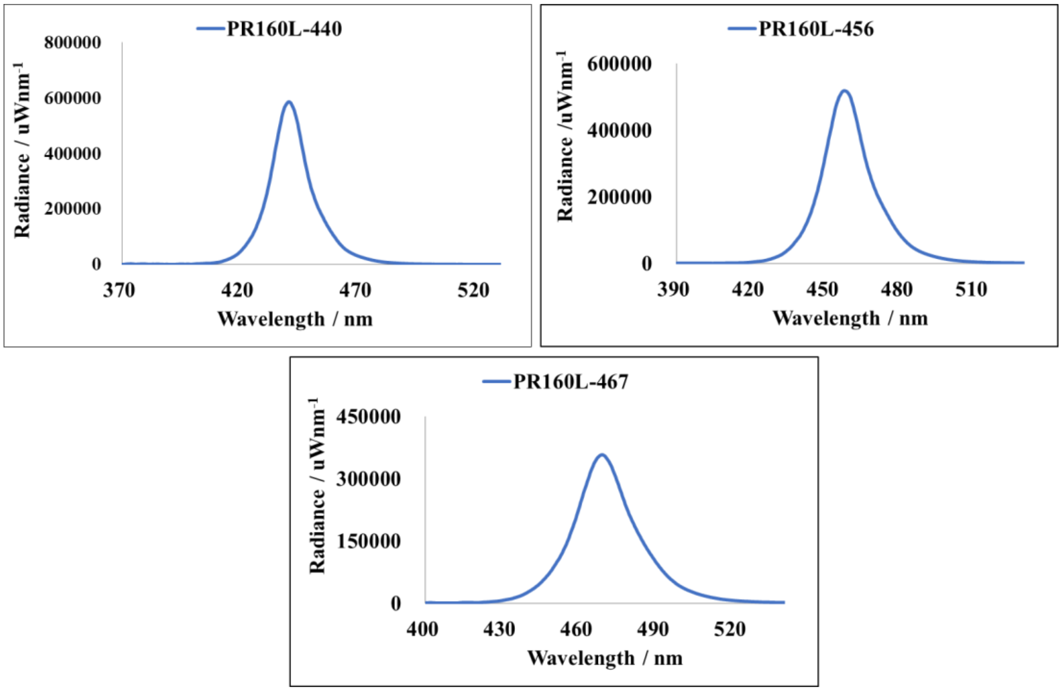

Figure S44 Emission spectra of light sources (Kessil PR-160L).

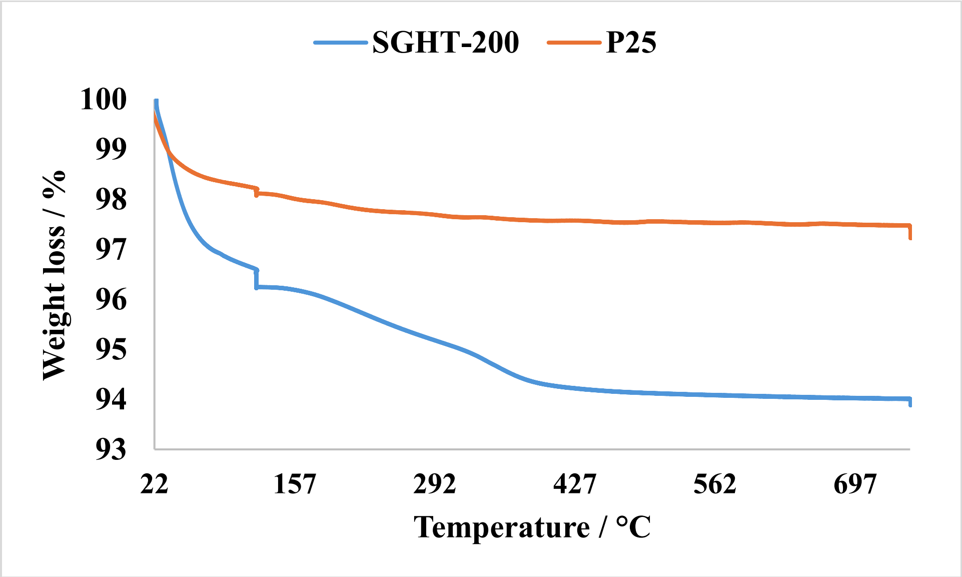

Figure S45 Thermogravimetric analysis of SGHT-200 and commercial titania P25.

2-(2-methoxyphenoxy)-1-(4-methoxyphenyl)ethanone

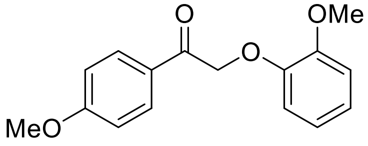

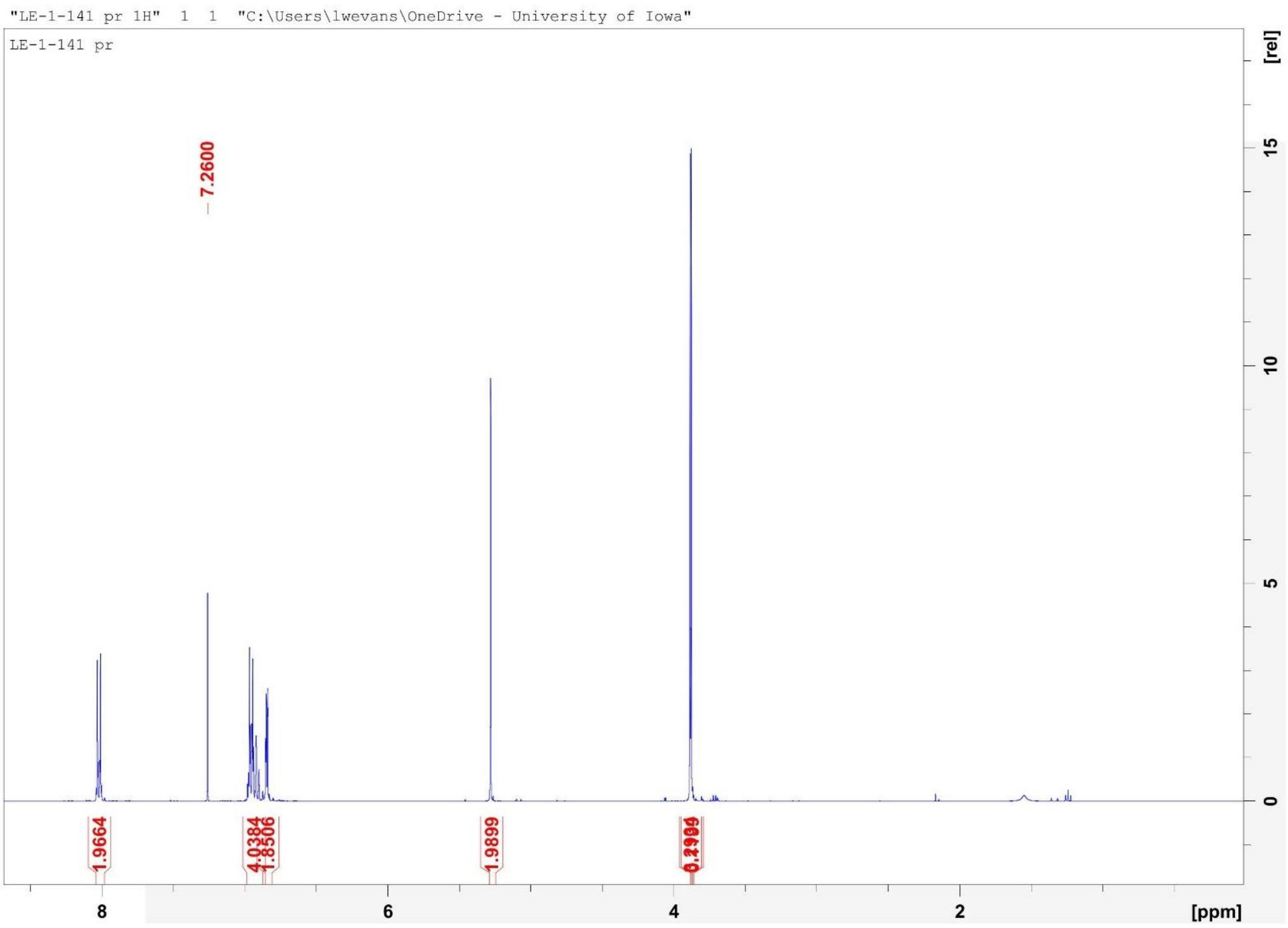

Figure S46 ^{1}H NMR spectra for 2-(2-methoxyphenoxy)-1-(4-methoxyphenyl)ethenone.

2-phenoxy-1-phenylethanone

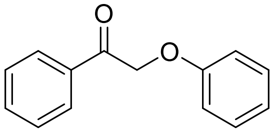

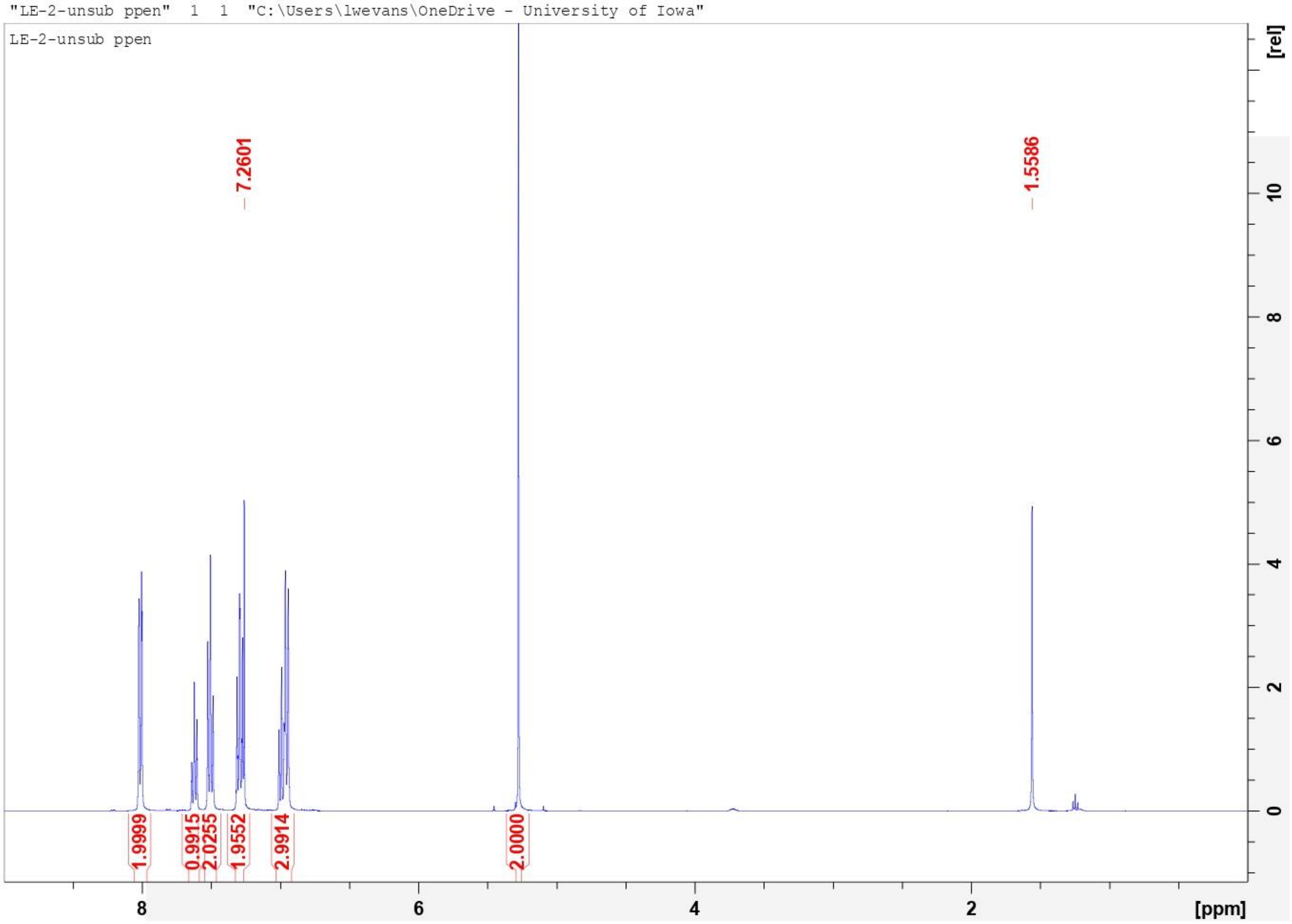

Figure S47 ^{1}H NMR spectra for 2-phenoxy-1-phenylethanone.

2-(2-methoxyphenoxy)-1-phenylethanone

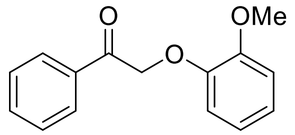

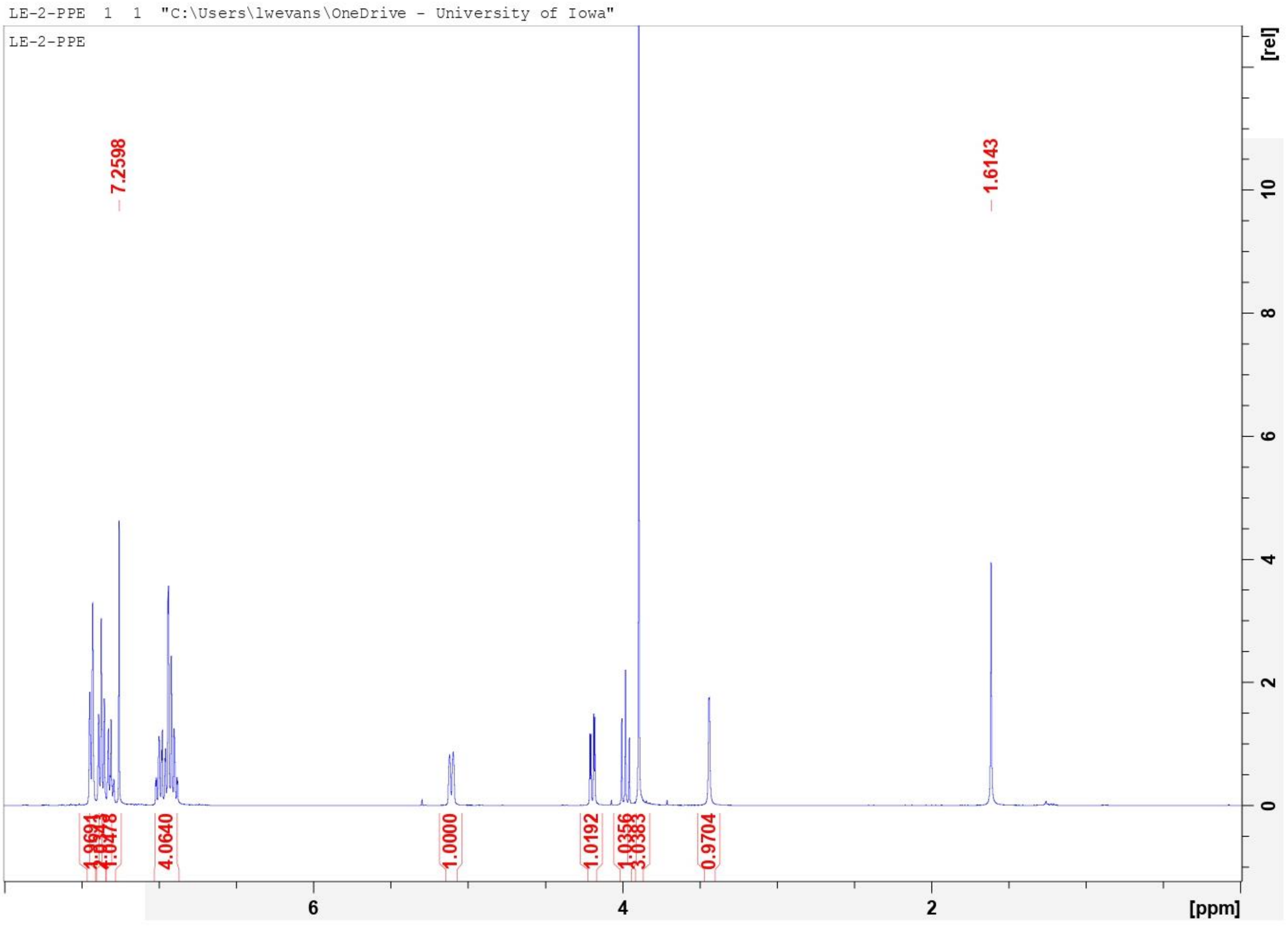

Figure S48 ^{1}H NMR spectra for 2-(2-methoxyphenoxy)-1-phenylethanone.

References

(1) Nichols, J. M.; Bishop, L. M.; Bergman, R. G.; Ellman, J. A. Catalytic C−O Bond Cleavage of 2-Aryloxy-1-Arylethanols and Its Application to the Depolymerization of Lignin-Related Polymers. *J Am Chem Soc* **2010**, *132* (36), 12554–12555. https://doi.org/10.1021/ja106101f.

(2) Luo, N.; Wang, M.; Li, H.; Zhang, J.; Hou, T.; Chen, H.; Zhang, X.; Lu, J.; Wang, F. Visible-Light-Driven Self-Hydrogen Transfer Hydrogenolysis of Lignin Models and Extracts into Phenolic Products. *ACS Catal* **2017**, *7* (7), 4571–4580. https://doi.org/10.1021/acscatal.7b01043.

(3) Pitre, S. P.; McTiernan, C. D.; Vine, W.; DiPucchio, R.; Grenier, M.; Scaiano, J. C. Visible-Light Actinometry and Intermittent Illumination as Convenient Tools to Study Ru(Bpy)3Cl2 Mediated Photoredox Transformations. *Sci Rep* **2015**, *5* (1), 16397. https://doi.org/10.1038/srep16397.

(4) Khan, A.; Goepel, M.; Kubas, A.; Łomot, D.; Lisowski, W.; Lisovytskiy, D.; Nowicka, A.; Colmenares, J. C.; Gläser, R. Selective Oxidation of 5-Hydroxymethylfurfural to 2,5-Diformylfuran by Visible Light-Driven Photocatalysis over In Situ Substrate-Sensitized Titania. *ChemSusChem* **2021**, *14* (5), 1351–1362. https://doi.org/10.1002/cssc.202002687.

(5) He, Y.-T.; Kang, D.; Kim, I.; Hong, S. Metal-Free Photocatalytic Trifluoromethylative Pyridylation of Unactivated Alkenes. *Green Chemistry* **2018**, *20* (22), 5209–5214. https://doi.org/10.1039/C8GC02782J.
